# Optimizing PSMA-617-based inhibitors through charged linker modifications: Insights into structure-activity relationships

**DOI:** 10.7150/thno.118972

**Published:** 2026-01-01

**Authors:** Nicolas M. Geis, Yvonne Braunwarth, Philipp T. Meyer, Matthias Eder, Ann-Christin Eder

**Affiliations:** 1 Department of Nuclear Medicine, University Medical Center Freiburg, Faculty of Medicine, University of Freiburg, Hugstetter Str. 55, 79106 Freiburg, Germany.; 2 Division of Radiopharmaceutical Development, German Cancer Consortium, Partner Site Freiburg, Hugstetter Str. 55, 79106 Freiburg, Germany, and German Cancer Research Center, Im Neuenheimer Feld 280, 69120 Heidelberg, Germany.; 3 Faculty of Biology, University of Freiburg, 79104 Freiburg, Germany.

**Keywords:** PSMA, prostate cancer, pharmacokinetic profile, charged linker, linker-modification

## Abstract

**Rationale:** The introduction of Pluvicto^®^ ([^177^Lu]Lu-vipivotide tetraxetan; [^177^Lu]Lu-PSMA-617) marks a milestone in radioligand therapy (RLT) for PSMA-positive metastatic castration-resistant prostate cancer (mCRPC). While dose escalation of [^177^Lu]Lu-PSMA-617 and alpha-emitting agents like [^225^Ac]Ac-PSMA-617 improves efficacy, it is limited by dose-dependent toxicity in critical organs, including kidneys, bone marrow and salivary glands. Modifications of the linker region in PSMA inhibitors have been proven to highly influence the pharmacokinetic profile. The utilization of charged linker moieties resulted in clinically used PSMA-targeting radiotracers such as [^18^F]PSMA-1007. This study explores histidine and/or glutamic acid-modified variants of PSMA-617 to investigate their effects on pharmacokinetic properties.

**Methods:** Based on the core structure of PSMA-617, eleven novel PSMA-targeting inhibitors were synthesized by introducing histidine and/or glutamic acid moieties at three positions within the linker region. Compounds were radiolabeled with [^68^Ga]Ga^3+^ and [^177^Lu]Lu^3+^ to assess their chemical and stability properties. Biological activity was evaluated in competitive cell binding and internalization assays with PSMA-expressing LNCaP cells. Dynamic and static small-animal PET imaging studies were conducted with the ^68^Ga-labeled inhibitors in LNCaP bearing BALB/c nu/nu xenografts to investigate their pharmacokinetic profiles.

**Results:** Precursors of linker-modified PSMA inhibitors presented high radiochemical purities (RCPs) for the complexation reactions with both radionuclides (>94%). ^68^Ga-labeled compounds demonstrated significantly lower lipophilicity (ranging from -3.4 to -3.9) compared to the reference compound [^68^Ga]Ga-PSMA-617 (-2.8 ± 0.3). Substantial effects on the affinity to PSMA were observed depending on the position and nature of modification (IC_50_ ranging from 10.40 ± 2.94 nM to 78.6 ± 44.1 nM). Modification with glutamic acid adjacent to the chelator resulted in a two-fold increase in affinity, while variants containing histidine and glutamic acid led to significant improvements in cell surface binding and internalization (*p* < 0.05). Dynamic small-animal PET scans with the novel ^68^Ga-labeled variants revealed an improved accumulation in LNCaP xenograft tumors (SUV_1 h_: 0.21 ± 0.05 to 1.32 ± 0.08 g/ml) accompanied by a fast clearance from the kidneys and background tissue within the initial 60 min. Static PET scans 2 h p.i. confirmed a high tumor uptake and a rapid renal excretion.

**Conclusion:** The introduction of histidine and/or glutamic acid moieties into the linker region of PSMA-617 resulted in measurable changes in pharmacokinetic properties both *in vitro* and *in vivo*. While some modifications led to improved tumor-to-kidney ratios and favorable early-stage excretion, it remains challenging to predict clinical off-targeting effects like salivary gland uptake. This study provides important insights into the structure-activity relationships of PSMA-617-related linker modifications and warrant additional investigation, including mechanistic and translational studies, to more accurately evaluate their therapeutic potential.

## Introduction

Prostate cancer remains one of the most common malignant diseases for men in the western world. Novel treatment strategies need to be developed, particularly for the increasing number of patients diagnosed with distant metastases of prostate cancer, as the overall survival rate after 5 years with the current standard treatment is only 32% 1. In response to the high demand for innovative therapeutic approaches, the clinical implementation of radiopharmaceuticals targeting the prostate-specific membrane antigen (PSMA) emerges as a promising approach. PSMA is a transmembrane carboxypeptidase characterized by its selective upregulation in the majority of prostate carcinomas, comprising localized lesions, osseous metastatic sites and malignant lymph nodes 2,3.

The recent approval of Pluvicto^®^ ([^177^Lu]Lu-vipivotide tetraxetan; [^177^Lu]Lu-PSMA-617) by the FDA and EMA represents the first radioligand therapy (RLT) for the treatment of PSMA-positive mCRPC 4. The findings of the prospective, multicenter Phase III VISION trial of [^177^Lu]Lu-PSMA-617 revealed a prolonged imaging-based progression-free survival and overall survival in individuals with advanced PSMA-positive mCRPC 5.

The use of beta-emitting [^177^Lu]Lu-PSMA-617 and, in particular, the potentially more effective alpha-emitting [^225^Ac]Ac-PSMA-617 is limited by adverse effects such as salivary gland irradiation 6,7. Xerostomia as a result of the high salivary gland uptake may represent a dose limiting factor especially with alpha emitters 8. In addition to close monitoring of the blood counts and the kidney function to minimize side effects, various strategies were tested to reduce salivary gland damage. However, none of these approaches have resulted in satisfactory outcomes for the patients, including external cooling, botulinum toxin injections, displacement strategies, radioprotectors and novel compounds 9-11. Thus, there is a high clinical demand for further innovation to improve the pharmacokinetic profile of PSMA-targeting radiopharmaceuticals and reduce side effects.

The majority of PSMA-targeting inhibitors exhibit a tripartite structure comprising a PSMA binding motif, a linker region and a chelator, with the linker region being crucial for the modulation of the biological activity 12. In the preclinical development of the chemical structure of PSMA-617, modifications in the linker region were a key factor improving the compound for today's broad clinical application 13. Among the various lipophilic building blocks within the linker of the PSMA inhibitor series, PSMA-617 showed the best combination of activity in tumor tissues and renal excretion with its linker consisting of 3-(2-naphthyl)-alanine (2NaI) and trans-4-(aminomethyl)cyclohexane carboxyamide (ChX).

In recent years, great efforts have been dedicated to enhancing the pharmacokinetic properties of PSMA-617 analogues through refinements within the linker region. Examples include the substitution of 2NaI with various aromatic amino acids, the introduction of albumin binding moieties or the addition of both natural and unnatural amino acids 14-16.

**Figure 1:**
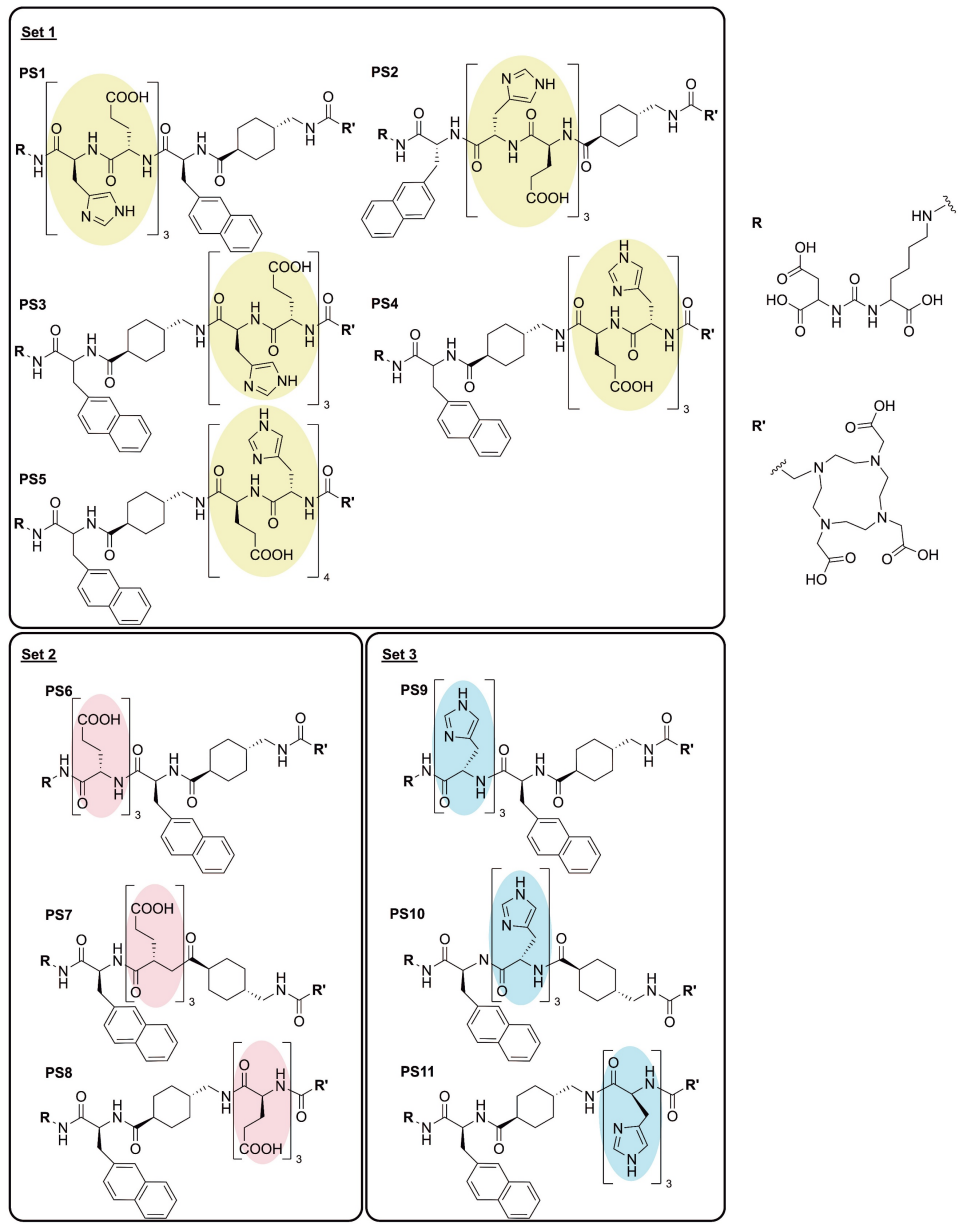
Overview of the linker modified PSMA-targeting inhibitors with glutamic acid (red, set 2), histidine (blue, set 3) and a combination of both (yellow, set 1).

To gain deeper insights into the effects of the introduction of charged amino acids into the linker region of PSMA-targeting tracers, we present an investigation of the development and evaluation of 11 novel radioligands derived from the chemical structure of PSMA-617 (Figure 1). While maintaining the inherent linker elements consisting of 2NaI and ChX, units of glutamic acid (E), histidine (H) and a combination of both were introduced into the three potential positions within the linker, located between the PSMA binding motif and 2NaI (P_1_), between the linker amino acids 2NaI and ChX (P_2_) and between ChX and the chelator DOTA (P_3_). Previous studies by Eder et al. have explored the positive effects of selected amino acid modifications and their number of insertions in HBED-CC (*N,N'*-bis(2-hydroxybenzyl)ethylenediamine-*N,N'*-diacetic acid) bearing compounds adjacent to the chelator 17. The present study further investigates the influence of a series of modifications within the structure PSMA-617, focusing on the impact of triplets and quartets of histidine and/or glutamate and their position in a therapeutic applicable compound.

## Methods

General Information of the chemicals, solvents, methods and instruments can be found in the SUPPLEMENTARY INFO (S1. General Info and S2. Supplementary Methods).

### Chemical synthesis

Solid-phase synthesis of the eleven modified variants of PSMA-617 comprising a set of charged amino acid linker modifications was performed similarly to previous published protocols (S2. Supplementary Methods, Figure S1 - S2) 13. The resin with the glutamate-urea-lysine binding motif (EuK; 0.1 mmol) was pre-swollen in dry dimethylformamide (DMF) for further synthesis. Relative to the resin, 4.00 equivalents of the first Fmoc-protected building block with 3.92 equivalents of 1-[Bis(dimethylamino)methylene]-1H-1,2,3-triazolo[4,5-b]pyridinium 3-oxide hexafluorophosphate (HATU) in the presence of 4.00 equivalents of *N,N*-diisopropylethylamine (DIPEA) in dry dimethylformamide (DMF) were used. The resulting solution was added to the pre-swollen resin and was agitated for 1 h at 36 °C.

Subsequently, selective removal of the Fmoc-protecting group from the added building block through washing with piperidine in DMF (20%) resulted in the intermediate product, without separate monitoring after each individual amino acid coupling. This process was repeated with 4.00 equivalents of a second, third etc. Fmoc-protected building block in dry DMF, with each reaction followed by Fmoc-protecting group deprotection as previously described 13. In the last step, 4.00 equivalents of the chelator 2-(4,7,10-tris(2-(*t*-butoxy)-2-oxoethyl)-1,4,7,10-tetraazacyclododecan-1-yl)acetic acid (DOTA-tris(*t*Bu)ester) were activated with 3.92 equivalents of HATU and 4.00 equivalents of DIPEA in DMF and added to the Fmoc-deprotected intermediate product to be mixed for 2.5 h at 36 °C.

To obtain the final product, the series of PSMA inhibitors PS1 - PS11 were subsequently cleaved from the resin by agitation with a cleavage cocktail consisting of trifluoroacetic acid (TFA), triisopropylsilane, and water (1 ml; 95:2.5:2.5) for 2 h. The product was precipitated in diethyl ether and purified with reverse phase flash chromatography (S2. Supplementary Methods).

### Radiolabeling

For ^68^Ga-labeled compounds, precursor peptides (2 or 5 nmol in 40 µl 1 M 2-[4-(2-Hydroxyethyl)piperazin-1-yl]ethane-1-sulfonic acid (HEPES) buffer, pH 4.0) were combined with [^68^Ga]Ga^3+^ eluate (40 µl, 30 - 60 MBq). The pH was adjusted to 4.2 using 30% sodium hydroxide (NaOH), followed by incubation at 95 °C for 15 min. Labeling for competitive cell binding experiments with [Glu-urea-Lys(Ahx)]_2_-HBED-CC (PSMA-10, ABX, Germany) was performed with the precursor (1.5 nmol in 40 µl 1M HEPES buffer, pH 4.0) and [^68^Ga]Ga^3+^ eluate (40 µl, 30 - 60 MBq). The pH was adjusted to 4.2 using 30% NaOH, followed by incubation at 95 °C for 10 min.

Radiolabeling with [^177^Lu]Lu^3+^ was completed by the addition of precursor peptides PS1 - PS11 (2 - 5 nmol in 50 µl 0.1 M HEPES buffer, pH 7.0) and [^177^Lu]Lu^3+^ in 0.04 M hydrochloric acid_(aq.)_ (3 µl, 30 - 50 MBq). The reaction mixture was heated to 95 °C for 15 min.

The radiochemical purity (RCP) was determined by analytical radio-HPLC (S2. Supplementary Methods).

### Partition coefficient (logD)

To determine the lipophilicity expressed as distribution coefficient (log*D*), a two-phase system of *n*-octanol (500 µl) and phosphate buffered saline (PBS) (490 µl) at pH 7.4 was used (n = 4). Compounds were radiolabeled with [^68^Ga]Ga^3+^ and the mixture diluted to a concentration of 0.25 µM in PBS. 10 µl of the 0.25 µM dilution were added to the *n*-octanol / PBS mixture. After extensive mixing, the 2-phase system was centrifuged for 10 min at 14,000 rpm and 4 °C. Two samples from each phase (100 µl) were taken and measured in a γ-gamma counter (PerkinElmer, Waltham, USA). The decimal logarithm of the ratio of the concentration of a compound in the *n*-octanol phase to its concentration in the aqueous phase resulted in the desired log*D* values. The data is presented as the mean value with the standard deviation (SD).

### Serum stability

Incubation in human and murine serum (BioIVT, United Kingdom) was used to determine the stability of the described precursors. First, the substances were labeled with [^177^Lu]Lu^3+^ and added to 100 µl of pre-warmed serum (0.5 nmol, 3 - 10 MBq). The reaction mixture was incubated at 37 °C and 10 µl samples were taken at regular intervals (0, 6, 24, 48, 72 h). After precipitation of the proteins by the addition of 30 µl of acetonitrile, the probes were centrifuged for 2 min at 14,000 rpm and the stability determined by analytical radio-HPLC of the lysate.

### *In vitro* studies

The PSMA-positive human cell line LNCaP (human lymph node carcinoma of the prostate; ATCC CRL-1740) was cultivated adherently at 37 °C and humidified 5% CO_2_ with RPMI-1640 (Thermo Fisher Scientific, Germany) supplemented with 10% fetal bovine serum (Sigma-Aldrich, Germany), 1% sodium pyruvate (Life Technologies, Germany) and 1% penicillin/streptavidin (Thermo Fisher Scientific, Germany). A mixture of trypsin/ethylenediaminetetraacetic acid (0.05% / 0.02%; Thermo Fisher Scientific, Germany) was used for cell harvesting (last authentication: April 2024).

### Competitive cell binding (IC_50_)

To determine the binding affinity of the investigated compounds, competitive cell binding studies were performed with ^68^Ga-labeled PSMA-10. Dilution series of twelve concentrations in medium were prepared from the precursor peptides PS1 - PS11 including concentrations of 0, 0.5, 1, 2.5, 5, 10, 25, 50, 100, 500, 1,000, and 5,000 nM. The assay was performed on 96-well filter plates (Merck, Darmstadt, Germany). Medium (100 µl per well) was added before the experiment and incubated for 1 h at room temperature to block non-specific binding. The medium was removed and the diluted compounds were incubated with [^68^Ga]Ga-PSMA-10 (0.8 nM) and LNCaP cells (10^5^ cells/well) for 45 min at room temperature (total volume 100 µl/well). Cells were washed with PBS buffer (2× 100 µl, 1× 200 µl) over a MultiScreen 96-well vacuum filtration system (Merck Millipore, Billerica, USA). The filters containing the retained cells were punched out and the radioactivity was quantified using a γ-counter. By applying a non-linear regression algorithm (GraphPad Prism 8.0.1., Boston, USA), the specific inhibitory concentration of 50% (IC_50_) could be determined. The experiment was repeated at least three times per compound and the data presented as IC_50_ ± standard deviation.

### Internalization studies

Internalization assays were performed on poly-L-lysine coated 24-well cell culture plates. The day before the experiment, LNCaP cells (10^5^ cells/well) were harvested and seeded to the wells. The cells were incubated with the ^68^Ga-labeled compounds (250 µl, 30 nM, diluted in RPMI medium) for 45 min at 37 °C. To determine the specific internalization, half of the wells were simultaneously incubated additionally with the highly potent PSMA inhibitor 2-(phosphonomethyl)pentanoic acid (2-PMPA, 500 µM, Sigma-Aldrich, Germany). The medium was then removed and the cells washed with ice-cold PBS buffer (3 × 1 ml). To remove surface bound compounds, ice-cold glycine buffer (2 × 0.5 ml, 50 mM, pH 2.8) was added to the wells and collected after 5 min in a combined fraction. After washing once with PBS (1 ml), NaOH (0.5 ml, 0.3 M) was added to the cells for lysation. Collected surface-bound, internalized fractions and standards from the stock solutions (25 µl) were measured in a γ-counter. Data was normalized and presented as percentage of the initially added radioactivity bound to 10^5^ cells [%IA/10^5^ cells].

### *In vivo* studies

The *in vivo* experiments were performed in adherence to the general animal welfare regulations of Germany (G18/04) and the institutional guidelines for the care and use of animals. 7- to 8-week-old male BALB/ c nu/nu mice were purchased from Charles River (Sulzfeld, Germany) or Janvier Labs (Le Genest-Saint-Isle, France). To establish tumor xenografts, 5 × 10^6^ cells of LNCaP (in 50% Martigel; Becton Dickinson, Germany) were subcutaneously injected into the right flank of each mouse. For imaging, mice were anaesthetized with isoflurane (2% isoflurane) and the ^68^Ga-labeled compounds were injected into a tail vein (9 - 13 MBq; 500 pmol in 100 µl). During the first 60 min., a dynamic positron emission tomography (PET)-scan with a small animal PET-scanner (microPET Focus 120; Concorde Microsystems Inc., Knoxville, USA) was performed. Afterwards, a static scan was conducted at 1 h p.i. The mice were allowed to wake up and another scan was performed 2 h p.i. For testing each compound, one exemplary animal was used. The PET sinograms were reconstructed using a 2-dimensional ordered subset expectation maximization (OSEM) algorithm. For quantification of radioactivity uptake, small volumes-of-interest (VOIs) were drawn manually using PMOD 3.704 (PMOD Technologies, Switzerland) to determine the standardized uptake values (SUV_max_).

### Statistical analysis

Experiments were conducted with a minimum of three independent replicates, with the exception of the PET imaging study with a sample size of one. Quantitative data are represented as the mean ± standard deviation. The statistical analysis was carried out using GraphPad Prism 8.0.1. (Boston, USA), Statistical significance was assessed through an unpaired, two-tailed t-test. P-values of less than 0.05 were considered statistically significant.

## Results

### Synthesis, analytics and radiolabeling

Each of the 11 novel PSMA inhibitors was synthesized according to known chemical synthesis procedures via on-resin solid phase peptide synthesis. Charged spacers were successfully introduced into the original chemical structure of PSMA-617 using Fmoc-based solid phase chemistry. All compounds were purified by flash chromatography, yielding in chemical purities exceeding 95% (Table 1). Additional analytical data of the final compounds PS1 - PS11 can be found in Figure S3 - S13. Synthesized inhibitors were grouped into linker modifications with glutamate and histidine as Set 1 (PS1 - PS5), linker-modifications with glutamate as Set 2 (PS6 - PS8) and linker-modifications with histidine as Set 3 (PS9 - PS11) (Figure 1).

After synthesis, the radiotracers were radiolabeled with gallium-68 and lutetium-177 resulting in radiochemical yields of greater than 94% for the ^68^Ga-labeled and greater than 96% for the ^177^Lu-labeled compounds (Table 1, Figure S14 - S25).

### *In vitro* stability and partition coefficient

**Figure 2:**
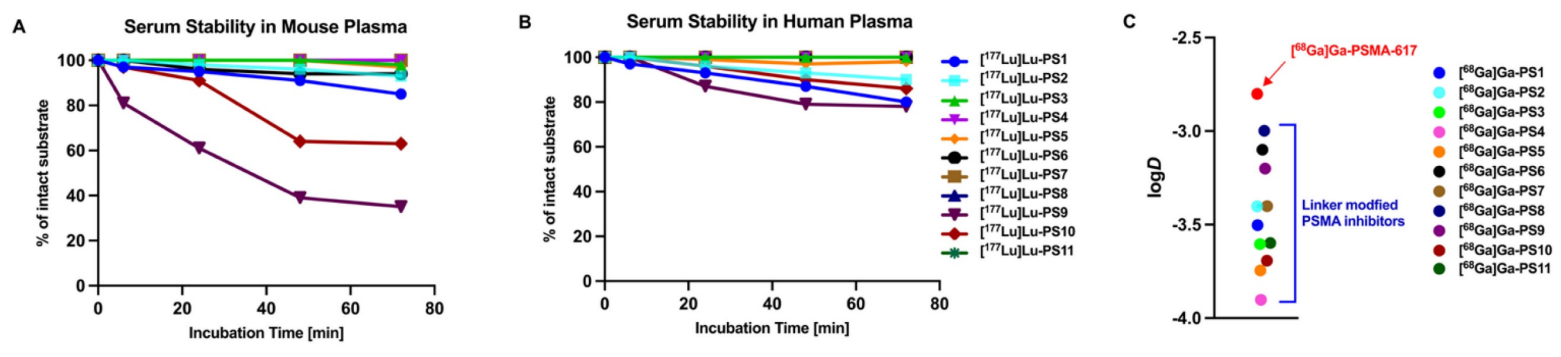
(A) Serum stability of the ^177^Lu-labeled compounds in Mouse Plasma. (B) Serum stability of the ^177^Lu-labeled compounds in Human Plasma. (C) Partition Coefficient (log*D*) at pH 7.4 in octanol/PBS of the ^68^Ga-labeled linker-modified compounds and PSMA-617.

For further *in vitro* analysis, the ^177^Lu-labeled compounds were incubated in human and mouse plasma and tested for degradation via HPLC at intervals of 0, 6, 24, 48 and 72 h. Most PSMA inhibitors incubated in mouse serum demonstrated stability exceeding 72 h, with degradation levels below 20% (Figure 2A, Table S2). In contrast, [^177^Lu]Lu-PS9 and [^177^Lu]Lu-PS10 exhibited a noticeable decomposition of nearly 40% after 24 h for [^177^Lu]Lu-PS9 and only 64% intact [^177^Lu]Lu-PS10 after 48 h. Incubation in human plasma resulted in overall better stability for the labeled inhibitors, with more than 75% of intact compound observed at the final time point for all tested compounds (Figure 2B, Table S3).

The Partition Coefficient (log*D*) was determined in an octanol/PBS mixture at pH 7.4 using the ^68^Ga-labeled compounds (Figure 2C). [^68^Ga]Ga-PSMA-617 exhibited the lowest hydrophilicity, displaying a log*D* value of -2.8 ± 0.3. Novel compounds containing histidine and glutamate moieties demonstrated a significantly higher hydrophilicity ranging from -3.4 to -3.9 as compared to [^68^Ga]Ga-PSMA-617 (each *p* < 0.05, Table S1). Moreover, linker modifications involving glutamate ([^68^Ga]Ga-PS6 - [^68^Ga]Ga-PS8) and histidine ([^68^Ga]Ga-PS9 - [^68^Ga]Ga-PS11) also exhibited an increased hydrophilicity compared to the parental tracer. Notably, the set of histidine-modified substances showed lower log*D* values on average compared to the glutamate-modified substances.

### Specific cell binding and internalization

All tested compounds exhibited a specific affinity to PSMA with a low nanomolar IC_50_ using LNCaP cells (IC_50_ ranging from 10.40 ± 2.94 nM to 78.6 ± 44.1 nM; Table 1). PSMA-617 was evaluated along the novel linker-modified compounds as an internal standard (21.77 ± 3.13 nM). The introduction of glutamate and/or histidine moieties resulted in a decrease of affinity for all the compounds except for PS8, which showed a significant higher affinity of 10.40 ± 2.94 nM (*p* = 0.004). Notably, the majority of increases in the IC_50_ values among the modified inhibitors did not demonstrate statistical significance, indicating a similar affinity comparable to PSMA-617.

**Figure 3:**
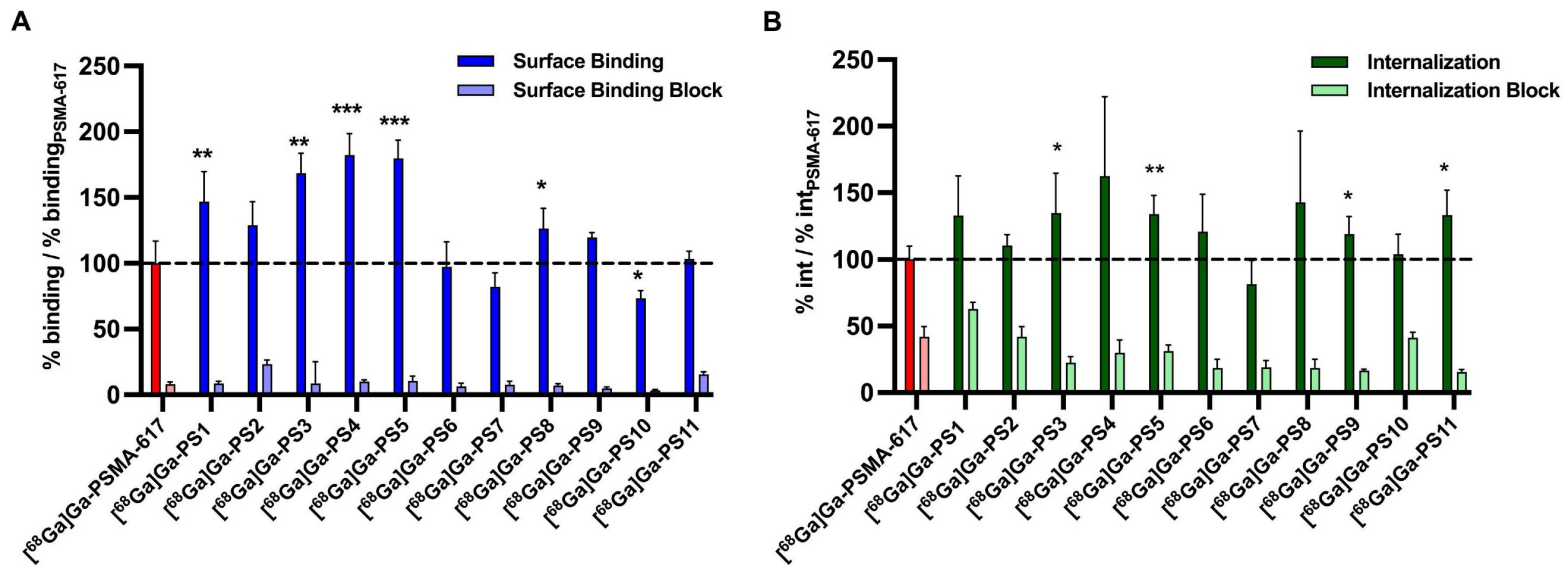
Surface binding (A) and internalization (B) of ^68^Ga-labeled PSMA inhibitors on PSMA-positive LNCaP cells after 45 min at 37 °C relative to the reference [^68^Ga]Ga-PSMA-617. (* : p < 0.05; ** : p < 0.01; *** : p < 0.001).

Regarding the surface binding of ^68^Ga-labeled compounds, results were normalized and specific binding to PSMA-positive LNCaP cells was demonstrated (Figure 3A, Table S5 - S6). Experiments of most compounds containing glutamate and histidine modifications (set 1) resulted in a significant increase of surface binding, more specific with [^68^Ga]Ga-PS1 (*p* = 0.0088), [^68^Ga]Ga-PS2 (*p* = 0.056), [^68^Ga]Ga-PS3 (*p* = 0.0011), [^68^Ga]Ga-PS4 (*p* = 0.0005) and [^68^Ga]Ga-PS5 (*p* = 0.0001). In set 2, only the introduction of glutamate units next to the chelator in [^68^Ga]Ga-PS8 led to a significant improved surface binding (*p* = 0.042) compared to [^68^Ga]Ga-PSMA-617. In contrast, in set 3 with histidine modifications, [^68^Ga]Ga-PS10 was the only compound with significantly reduced surface binding (*p* = 0.039). Histidine moieties next to the PSMA binding motif in [^68^Ga]Ga-PS9 and next to the chelator in [^68^Ga]Ga-PS11 did not result in significant changes as compared to the parental compound.

Significant higher internalization was found for the compounds with glutamate and histidine modifications [^68^Ga]Ga-PS3 (*p* = 0.0046) and [^68^Ga]Ga-PS5 (*p* = 0.0036) (Figure 3B, Table S5 and S7). The other compounds in set 1 did not show any significant changes. Furthermore, introduction of glutamate moieties in set 2 did not alter the internalization properties significantly. Regarding set 3, moieties next to the PSMA binding motif in [^68^Ga]Ga-PS9 (*p* = 0.042) and next to the chelator in [^68^Ga]Ga-PS11 (*p* = 0.014) led to significant improvements in internalization.

### *In vivo* evaluation

#### Dynamic PET imaging (0 - 60 min)

**Figure 4:**
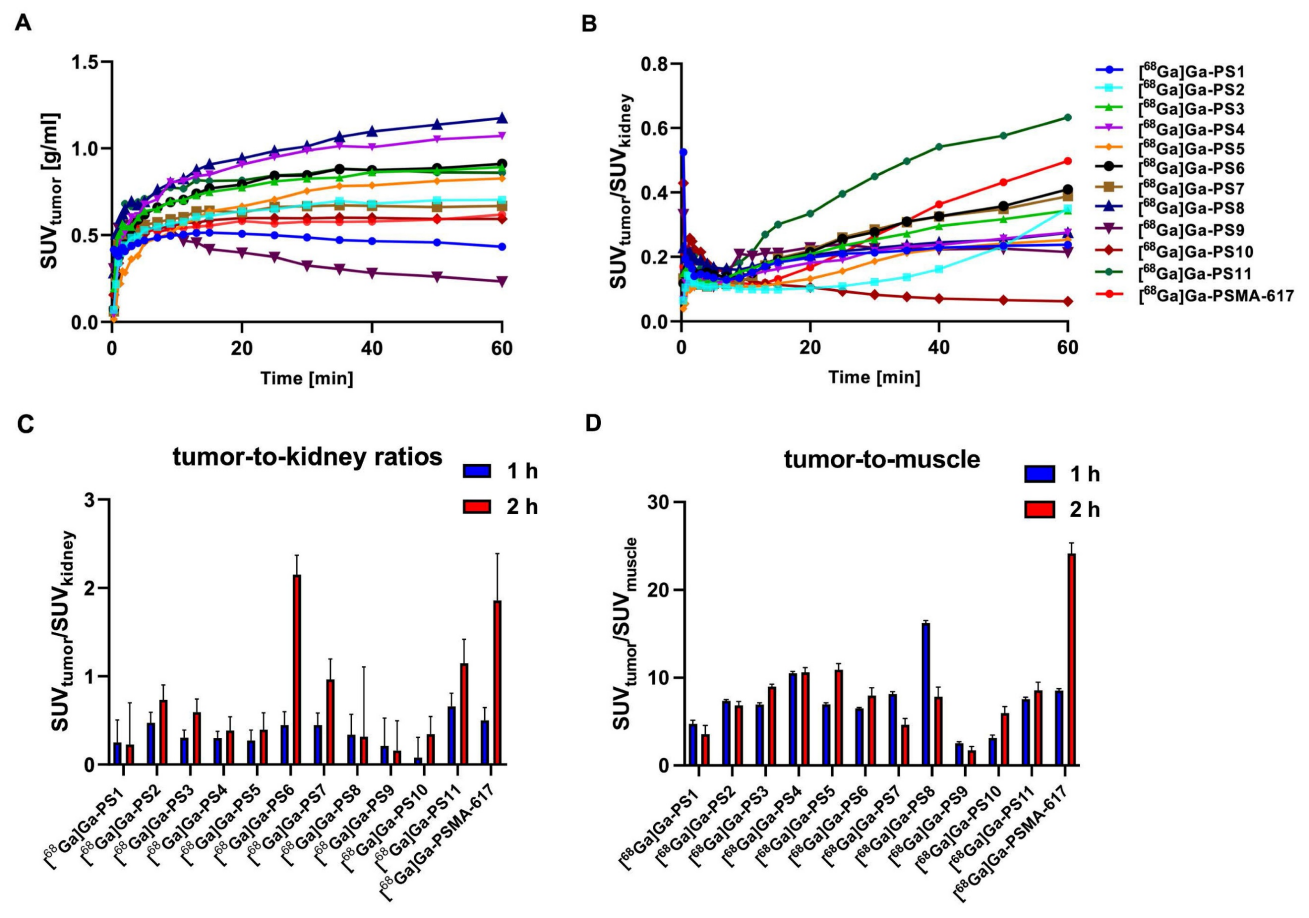
Tumor accumulation (A) and tumor-to-kidney-ratios (B) of the dynamic PET imaging 0 - 60 min. Tumor-to-kidney ratios (C) and tumor-to-muscle ratios (D) of the static small PET imaging at 1 h and 2 h p.i. in LNCaP xenograft-bearing BALB/c nu/nu mice after the injection of 0.5 nmol of the ^68^Ga-labeled compounds.

Dynamic small-animal PET imaging in LNCaP-bearing xenografts confirmed a fast clearance from non-target tissue with a strong tumor uptake and fast renal excretion for most of the linker-modified compounds within the first 60 min (Figure 4A - B, Figure S26 - S27).

Tumor accumulation in the dynamic PET scan (Figure 4A) was the highest for [^68^Ga]Ga-PS4 (SUV_1 h, tumor_ = 1.072 g/ml) and [^68^Ga]Ga-PS8 (SUV_1 h, tumor_ = 1.174 g/ml) in the tumor xenografts within the first 60 min, higher than [^68^Ga]Ga-PSMA-617 (SUV_1 h, tumor_ = 0.618 g/ml). Slightly slower tumor accumulation was observed in most of the remaining compounds. [^68^Ga]Ga-PS1 and [^68^Ga]Ga-PS9, however, showed a significant reduction of activity in the tumor within the first minutes after application.

After analysis of the tumor-to-kidney ratios (SUV_tumor_/SUV_kidney_), [^68^Ga]Ga-PSMA-617 (0.498 after 60 min) and [^68^Ga]Ga-PS11 (0.633 after 60 min) demonstrated the most favorable results in the dynamic study (Figure 4B). Interestingly, these two inhibitors also showed the fastest renal clearance beside [^68^Ga]Ga-PS9. The tumor-to-kidney ratios for the remaining compounds notably exhibited an inferior performance.

#### Static PET imaging - tumor accumulation

**Figure 5:**
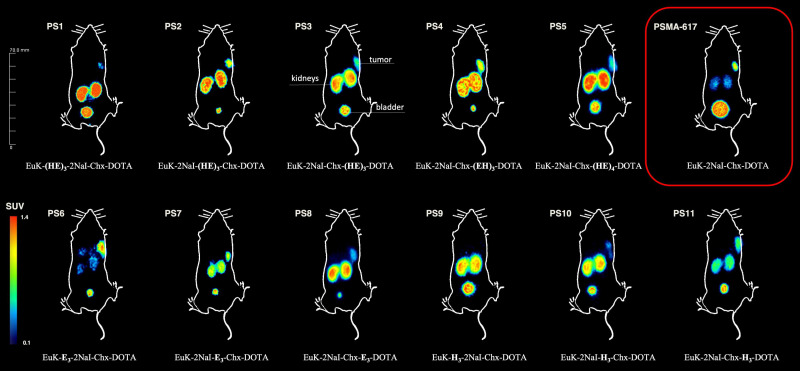
Maximum intensity projections (MIP's) 2 h p.i. of 0.5 nmol of the ^68^Ga-labeled compounds in LNCaP xenograft- bearing BALB/c nu/nu mice.

Evaluation of the tumor accumulation and retention after static small-animal PET imaging 1 h and 2 h p.i. (Figure 5, Figure S27, Table S8) confirmed the findings obtained from the dynamic PET imaging and *in vitro* experiments.

[^68^Ga]Ga-PS4 (SUV_1 h, tumor_ = 1.15 ± 0.04 g/ml) and [^68^Ga]Ga-PS8 (SUV_1 h, tumor_ = 1.32 ± 0.08 g/ml) showed the highest uptakes in the tumor at 1 h p.i. Within all sets, the majority of compounds showed higher tumor accumulation than the reference [^68^Ga]Ga-PSMA-617 (SUV_1 h, tumor_ = 0.63 ± 0.02 g/ml). Most of the compounds from set 1 demonstrated a good tumor retention at 2 h p.i. when compared to the 1 h p.i. scan and the reference [^68^Ga]Ga-PSMA-617 (SUV_2 h, tumor_ = 0.52 ± 0.16 g/ml). In particular, [^68^Ga]Ga-PS4 displayed the highest SUV-values for the tumor among all substances (1.07 ± 0.09 g/ml at 2 h p.i.). In contrast to the other inhibitors in set 1, [^68^Ga]Ga-PS1 showed a decrease in tumor accumulation starting at 1 h p.i. (SUV_1 h, tumor_ = 0.43 ± 0.06 g/ml, SUV_2 h, tumor_ = 0.30 ± 0.11 g/ml), representing the lowest SUV values of set 1 at these time points. Similarly, in set 2, [^68^Ga]Ga-PS8 revealed a substantial decrease after 2 h p.i. (SUV_2 h, tumor_ = 0.86 ± 0.39 g/ml). In contrast, the tumor SUVs of [^68^Ga]Ga-PS6 increased mainly between 1 h (SUV_1 h, tumor_ = 0.69 ± 0.07 g/ml) and 2 h p.i. (SUV_2 h, tumor_ = 0.85 ± 0.08 g/ml). In set 3, [^68^Ga]Ga-PS11 revealed the best tumor accumulation and retention compared to the other histidine-modified variants (SUV_1 h, tumor_ = 0.82 ± 0.07 g/ml, SUV_2 h, tumor_ = 0.88 ± 0.09 g/ml). Conversely, [^68^Ga]Ga-PS9 showed the lowest tumor uptake after 1 h (SUV_1 h, tumor_ = 0.21 ± 0.05 g/ml) and 2 h p.i. (SUV_2 h, tumor_ = 0.12 ± 0.03 g/ml) in the entire set of modified compounds.

#### Static PET imaging - tumor-to-organ ratios

Tumor-to-kidney (Figure 4C) and tumor-to-muscle (Figure 4D) ratios were compared to the results of [^68^Ga]Ga-PSMA-617 (1 h: 0.50 ± 0.14; 2 h: 1.86 ± 0.53).

In set 1, [^68^Ga]Ga-PS2 displayed the highest tumor-to-kidney-ratio after 1 h (0.47 ± 0.14) and 2 h p.i. (0.73 ± 0.14). Despite the favorable tumor accumulation with the introduction of (EH)_3_, compounds in set 1 showed no significant improvement regarding tumor-to-kidney ratios due to a slow washout from the kidneys, including the best performing compound [^68^Ga]Ga-PS4 (SUV_1 h, kidneys_ = 3.81 ± 0.15 g/ml and SUV_2 h, kidneys_ = 2.77 ± 0.20 g/ml). Similarly, the slow renal clearance of the glutamate-modified [^68^Ga]Ga-PS8 with a high dose in the kidneys (SUV_2 h, kidneys_ = 2.70 ± 0.89 g/ml) led to inferior tumor-to-kidney-ratios compared to the other compounds. Notably, [^68^Ga]Ga-PS6, which also incorporates glutamate modifications, exhibited the best improvement regarding tumor-to-kidney-ratio between 1 h (0.44 ± 0.15) and 2 h p.i. (2.15 ± 0.22), comparable to [^68^Ga]Ga-PSMA-617. In the histidine-modified set 3, [^68^Ga]Ga-PS9 and [^68^Ga]Ga-PS10 revealed notably lower tumor-to-kidney ratios at both time points compared to the majority of the other compounds. [^68^Ga]Ga-PS11 showed the best tumor-to-kidney ratio out of all tested inhibitors after 1 h (0.66 ± 0.15) and decent results after 2 h p.i. (1.15 ± 0.27). Due to the fastest clearance from the background and the good tumor retention, [^68^Ga]Ga-PSMA-617 revealed the most favorable tumor-to-muscle ratio 2 h p.i. (24.13 ± 1.22). [^68^Ga]Ga-PS4 and [^68^Ga]Ga-PS8 exhibited the best tumor-to-muscle ratios at the early timepoint of 1 h p.i. (10.52 ± 0.20 and 16.23 ± 0.27), similarly to the superior tumor accumulation (Figure 4D).

## Discussion

Analysis of the current field of PSMA inhibitors highlights the potential for improved radiopharmaceuticals with advantages in tumor uptake and clearance profiles 18,19. Considering the development of the chemical structure of PSMA-617, alternations of the linker region between the PSMA binding motif and the chelator were crucial in achieving the necessary improvements in its pharmacokinetic profile, allowing for clinical translation 13. Furthermore, introducing charged moieties like histidine and glutamate into the linker region resulted in substantial improvements of the organ distribution 20-22. Previous work demonstrated the benefits of introducing (HE)_3_ tags into imaging tracers targeting PSMA-expressing prostate tumors 17,23. The present study extends the concept of introducing charged amino acids into the linker of PSMA inhibitors to the therapeutically applicable PSMA-617. Additionally, further variation of the amino acids and altering the position of the modifications were investigated to gain deeper insights into the influence of the type and location of linker modifications in PSMA-targeting compounds. This systematic approach provides a more comprehensive understanding of structure-activity relationships than previous single-position studies.

**Figure 6:**
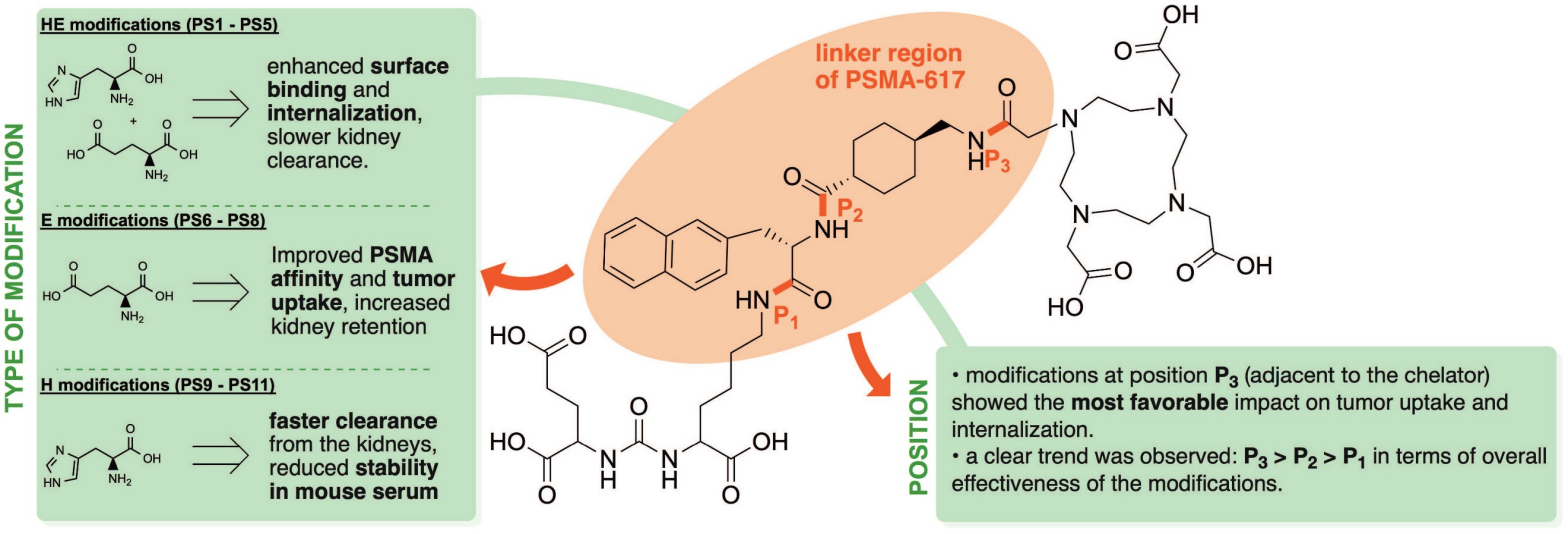
Summary of structure-activity relationships derived from experimentally observed trends in *in vivo* and *in vitro* experiments for PSMA-617 analogues with various linker modifications.

Based on these findings, we conducted a study aimed at further investigating the potential advantages of charged linker modifications in the therapeutically used PSMA inhibitor PSMA-617. A set of eleven novel compounds was synthesized, utilizing the chemical structure of PSMA-617. Units of glutamate and/or histidine were introduced into the three possible positions of the linker region of PSMA-617, while maintaining the original linker moieties of 2NaI and ChX. By the present approach we investigated both the influence of the type of modification and the influence exerted by the position of said modifications. A general summary of the trends found in this study regarding the position and type of modification is provided in Figure 6. Comparable to PSMA-617, employing DOTA as the chelator provided the option to label the novel compounds with therapeutically viable radionuclides like lutetium-177 or actinium-225. After successful chemical synthesis and radiolabeling, most compounds showed the required *in vitro* stability properties for further testing. The only substances exhibiting substantial degradation in mouse serum were the histidine-modified inhibitors [^177^Lu]Lu-PS9 and [^177^Lu]Lu-PS10. This is in line with numerous studies in the past observing higher degradation levels for peptide based drugs in mouse serum compared to human serum.^24^-^26^ Interestingly, the histidine-modifications in [^177^Lu]Lu-PS11 did not result in any degradation, potentially due to the position next to the bulky chelator DOTA. Furthermore, introducing basic amino acids like histidine and/or acidic amino acids like glutamate, caused a higher net charge of the modified compounds at neutral pH conditions. Linker-modified inhibitors with histidine and/or glutamate collectively presented a higher hydrophilicity compared to the original compound, PSMA-617. Specifically, compounds in set 1 contained a higher total count of charged amino acids in comparison to compounds in set 2 and set 3, resulting in significantly lower log*D* values.

Similar to PSMA-617, all novel compounds showed a low nanomolar affinity to PSMA-expressing LNCaP cells, with high specific binding and internalization. Regarding the position of the modifications, compounds with modifications inserted next to the chelator (P3) showed the lowest IC_50_ values within their set, whereas modifications next to the binding motif (P1) resulted in the highest IC_50_ values. Considering the type of modification, compounds with glutamate showed the highest affinity to PSMA- expressing cells, with PS8 being the only compound with a significantly lower IC_50_ value than PSMA-617. Compounds from set 1 with histidine and glutamate modifications presented the highest surface binding on LNCaP cells, which is in line with previous work on introducing HE units into the radiotracer [^68^Ga]Ga-PSMA-11 23. Modifications next to the chelator resulted in the greatest improvements in surface binding, with significant increases of nearly 2-fold for some compounds in set 1 and significant improvements for [^68^Ga]Ga-PS8 in set 2 compared to [^68^Ga]Ga-PSMA-617. Internalization was improved for all variants with modifications next to the binding motif (P1) or next to the chelator (P3). Interestingly, multiple histidine-containing compounds from set 1 and 3 performed considerably better in the internalization studies than [^68^Ga]Ga-PSMA-617. Consistent with the results of the surface binding, chelator-proximal modifications yielded significantly improved internalization, particularly evident for [^68^Ga]Ga-PS3, [^68^Ga]Ga-PS5 and [^68^Ga]Ga-PS11.

In summary, while the affinity and the internalization assays demonstrated clear trends in the sets independently, direct correlations between these parameters were limited. This discrepancy likely reflects the multifactorial processes involving additional factors beyond the primary PSMA-ligand interaction 27. The negatively charged cell surface - due to phospholipid headgroups and sialic acids and other negatively charged carbohydrate moieties on glycoproteins and glycolipids - may hinder the internalization or stable binding of glutamate-containing compounds, despite their high affinity. In contrast, histidine moieties could enhance electrostatic attraction and potentially promote more efficient clathrin-mediated internalization. Notably, the improved affinity observed for set 2 compounds suggests that combining glutamate and histidine in the linker may optimize both affinity and net charge for an optimal cellular uptake.

Results of the small-animal PET imaging supported the obtained data from the *in vitro* experiments. The compound with the best results in the affinity assays, more specifically the glutamate modified [^68^Ga]Ga-PS8, showed the highest accumulation in the PSMA-positive LNCaP tumor in PET imaging 1 h p.i. Similarly, Huang *et al.* reported positive effects by introducing negatively charged linkers into PSMA inhibitors 28. [^68^Ga]Ga-PS4 with its glutamate and histidine modifications performed most favorable regarding surface binding and internalization, and demonstrated improvement in tumor accumulation when compared to [^68^Ga]Ga-PSMA-617. Interestingly, both compounds contained modifications next to the chelator. For the histidine-modified compounds in set 3, the introduction of histidine moieties adjacent to the chelator in [^68^Ga]Ga-PS11 resulted in the highest tumor accumulation at all time points compared to the other histidine-modified variants. Most linker-modified compounds showed similar trends when comparing the *in vitro* and *in vivo* assays. Variants with a high internalization rate typically showed higher tumor uptakes. With respect to the tumor-to-kidney ratio, the dynamic PET imaging revealed histidine modified [^68^Ga]Ga-PS11 as the top compound at 1 h p.i., due to a fast kidney clearance. However, *in vitro* experiments in this study could not predict the *in vivo* properties of the renal clearance in a satisfactory manner. Inhibitors composing high accumulation in the tumor tended to elevated levels of activity in the kidneys. High expression levels of PSMA in the mouse kidneys might account for the increased activities, especially for compounds demonstrating high affinity towards PSMA and good internalization properties 29. Notably, variants with the highest tumor accumulation such as [^68^Ga]Ga-PS4 or [^68^Ga]Ga-PS8 demonstrated less favorable tumor-to-kidney ratios at the later time point due to the accumulation of activity in the kidneys. In contrast, previously described PSMA-targeting compounds with negatively charged linker demonstrated a rapid excretion through the kidneys and minimal non-specific binding 30. Analyzing the static PET scan at 2 h p.i. confirmed the importance of a fast kidney clearance. Glutamate modifications next to the PSMA binding motif in [^68^Ga]Ga-PS6 and the reference [^68^Ga]Ga-PSMA-617 showed the highest tumor-to-kidney ratios. These results contrast with typical findings, which suggest that top candidates for PSMA affinity and tumor accumulation exhibit the highest tumor-to-kidney and tumor-to-muscle ratios. In other compounds like [^18^F]PSMA-1007, glutamate units were introduced into the linker region to add negative charge to the chemical structure resulting in better *in vivo* properties 20. Our results could partly affirm these observations, although the importance of the position of linker modification was confirmed again. Similarly, compounds with high tumor accumulation displayed superior tumor-to-muscle ratios. Rapid clearance from the background and good tumor retention emerged as the crucial factors, resulting in the superior performance of [^68^Ga]Ga-PSMA-617 regarding the tumor-to-muscle ratio at the 2 h p.i. time point. With regard to the position of the modifications, no clear trends could be observed in terms of tumor-to-kidney and tumor-to-muscle ratios. Superior *in vivo* results of HE units in the linker region in the previous work with [^68^Ga]Ga-PSMA-11 were not observed in this study, especially at the later time point 23. In general, this study indicates that *in vitro* assays, more specifically binding affinity and internalization, can effectively predict *in vivo* tumor accumulation, especially at early time points p.i. However, predicting pharmacokinetic properties such as renal clearance and non-malignant tissues were not possible. As a result, potential advantages of modifications next to the chelator (P3) seen in the *in vitro* assays can only be transferred to a limited extent, emphasized by better *in vivo* results of some substances with modifications at the P1 position.

Rapid clearance of activity from the kidneys and non-malignant tissues confirmed the main advantage of the parental compound [^68^Ga]Ga-PSMA-617, particularly at later time points. However, it was shown, that charged linker modifications can improve *in vitro* and *in vivo* properties of PSMA inhibitors. [^68^Ga]Ga-PS4 and [^68^Ga]Ga-PS8 demonstrated a superior tumor uptake compared to [^68^Ga]Ga-PSMA-617 at 1 h and 2 h p.i. Additionally, histidine modifications next to the chelator in [^68^Ga]Ga-PS11 led to improved tumor-to-kidney ratios at earlier time points.

In summary, the study contributes to the growing body of literature on linker modifications in PSMA-targeting tracers. However, the majority of studies focused mainly on either modifications or substitution of the original linker of PSMA-617, resulting in only minimal improvements compared to the parent structure 16,31. The recently published crystal structure of the PSMA/PSMA-617 complex enables profound insights and a more targeted design of new tracers 32. Previous work of Huang et al. demonstrated the advantages of integrating highly negative linker elements into PSMA-targeting compounds 28,30. Promising results from a study with small changes in the existing linker units of PSMA-617 are unfortunately only insufficiently applicable due to the lack of a direct comparison with the original substance 33. Eder et al. demonstrated that the introduction of repeating histidine-glutamate motifs within the linker domain of PSMA-11-based tracers significantly improved tumor-to-background ratios by enhancing clearance from non-target tissues without influencing tumor uptake 17. In contrast, our translation of this design to PSMA-617 analogues, despite utilizing the same charged residues, resulted in modest improvements and did not fully replicate the magnitude of pharmacokinetic gains, highlighting the scaffold-dependent nature of linker modifications.

While our results demonstrate striking correlations between individual linker-region modifications and increased tumor uptake and *in vitro* results, the underlying molecular and structural mechanisms for such effects remain to be elucidated. Our structure-activity relationship conclusions are therefore based on emergent biological trends in our compound sets rather than direct evidence of alternative PSMA binding modes or routes of cellular entry. Previously mentioned studies suggested that charged or hydrophilic residues at linker positions can affect ligand affinity and pharmacokinetics by altering net charge, hydrophilicity, or interaction with the neighboring PSMA residues, whose at least partial explanation may be our findings. However, as our study did not include direct mechanistic assays or structural analyses, these structure-activity relationship observations should be regarded as preliminary and correlative and require additional experiments to validate the data.

Despite the promising results, several limitations should be acknowledged. Most importantly, *in vivo* PET imaging used a single animal per compound, limiting statistical significance and the assessment of biological variability. Also, all the experiments were performed in a single tumor model, and results may not generalize to other settings or to clinical practice. Furthermore, while mouse models are valuable for preclinical evaluation, they do not fully recapitulate some off-target effects apparent in humans, including salivary gland uptake. While our approach aims to reduce off-target uptake through enhanced clearance and altered pharmacokinetics, definitive evaluation of safety, especially in the salivary glands, will require clinical or other translational models.

## Conclusions

Modifications of the linker region of PSMA-617 analogues with charged moieties such as glutamate and/or histidine significantly influence both PSMA affinity and pharmacokinetic properties. The presented data provide valuable insights into structure-activity relationships, with clear intra-assay trends regarding the position and type of modification. Modifications at the position adjacent to the chelator (P3) were associated with the most promising effects. However, increased tumor uptake often correlated with elevated renal retention. In contrast, the introduction of histidine units at the same position (e.g., PS11) led to improved tumor-to-kidney ratios at early time points, likely due to enhanced renal clearance. Nonetheless, it remains challenging to predict the optimal position for the incorporation of new modifications, as the pharmacokinetics, especially at later time points, can only be predicted to a limited extent by *in vitro* assays. Overall, this study enhances our understanding of how charged linker modifications can be strategically employed. These findings can be employed to further optimize the pharmacokinetic profile of PSMA-617 by maximizing tumor uptake while minimizing off-target organ retention, especially in kidneys and non-target tissue.

## Supplementary Material

Supplementary methods, figures and tables.

## Figures and Tables

**Table 1:** Analytical data of the synthesized PSMA inhibitors and results of the radiochemical purity (RCP) with [^68^Ga]Ga^3+^ and [^177^Lu]Lu^3+^, Partition Coefficient (log*D*) determined in *n*-octanol/PBS at pH 7.4 and affinity against PSMA-positive LNCaP cells (IC_50_).

	Abbr.	Compound	M_calc._ m/z [g/mol]	ESI m/z [g/mol]	Chemical purity [%]	RCP ^68^Ga [%]	RCP ^177^Lu [%]	Partition Coefficient [log*D*_octanol/PBS_]*	IC_50_ [nM]
PSMA-617		EuK-NaI-ChX-DOTA	-	-	-	99	> 99	-2.7 ± 0.2	21.77 ± 3.13
	PS1	EuK-(HE)_3_-NaI-ChX-DOTA	1840.9	1843.6	> 95	94	99	-3.5 ± 0.2	55.98 ± 7.70
	PS2	EuK-NaI-(HE)_3_-ChX-DOTA	1840.9	1843.2	> 97	98	99	-3.4 ± 0.3	43.13 ± 9.63
Set 1	PS3	EuK-NaI-ChX-(HE)_3_-DOTA	1840.9	1843.5	> 99	99	> 99	-3.6 ± 0.3	37.0 ± 11.4
	PS4	EuK-NaI-ChX-(EH)_3_-DOTA	1840.9	1843.7	> 99	98	> 99	-3.9 ± 0.3	27.86 ± 6.93
	PS5	EuK-NaI-ChX-(HE)_4_-DOTA	2107.2	2109.8	> 97	95	96	-3.74 ± 0.05	46.7 ± 37.3
	PS6	EuK-E_3_-NaI-ChX-DOTA	1429.5	1432.2	> 99	97	> 99	-3.1 ± 0.3	28.55 ± 8.42
Set 2	PS7	EuK-NaI-E_3_-ChX-DOTA	1429.5	1432.2	> 99	>99	> 99	-3.3 ± 0.1	23.37 ± 9.70
	PS8	EuK-NaI-ChX-E_3_-DOTA	1429.5	1432.0	> 99	99	> 99	-3.0 ± 0.1	10.40 ± 2.94
	PS9	EuK-H_3_-NaI-ChX-DOTA	1453.6	1456.3	> 98	99	97	-3.2 ± 0.1	78.6 ± 44.1
Set 3	PS10	EuK-NaI-H_3_-ChX-DOTA	1453.6	1456.2	> 99	> 99	96	-3.7 ± 0.6	54.0 ± 20.9
	PS11	EuK-NaI-ChX-H_3_-DOTA	1453.6	1456.3	> 99	98	> 99	-3.6 ± 0.1	22.68 ± 6.47

*Partition Coefficient was determined with the ^68^Ga-labeled compounds.

## Abbreviations

%ID/g: injected dose per gram
2-PMPA: 2-(phosphonomethyl)pentanoic acid
2NaI: 3-(2-naphthyl)-alanine
ChX: trans-4-(aminomethyl)cyclohexane carboxyamide
DIPEA: *N,N*-diisopropylethylamine
DMF: dimethylformamide
DOTA: 2,2′,2′′,2′′′-(1,4,7,10-Tetraazacyclododecane-1,4,7,10-tetrayl)tetraacetic acid
E: glutamic acid
EuK: glutamate-urea-lysine
H: histidine
HATU: 1-[Bis(dimethylamino)methylene]-1H-1,2,3-triazolo[4,5-b]pyridinium 3-oxide hexafluorophosphate
HBED: *N, N'*-bis(2-hydroxybenzyl)ethylenediamine-N,N'-diacetic acid
HEPES: 2-[4-(2-Hydroxyethyl)piperazin-1-yl]ethane-1-sulfonic acid
LNCaP: human lymph node carcinoma of the prostate
mCRPC: metastatic castration-resistant prostate cancer
NaOH: sodium hydroxide
OSEM: ordered subset expectation maximization
p.i.: post injection
PBS: phosphate buffered saline
PET: positron emission tomography
PSMA: prostate specific membrane antigen
RCP: radiochemical purity
RLT: radioligand therapy
rpm: rounds per minute
SUV: standardized uptake value
TFA: trifluoroacetic acid
VOI: volume-of-interest
