# Supplementary material for: Optimizing PSMA-617-based inhibitors through charged linker modifications: Insights into structure-activity relationships
Source: Theranostics. 2026 Jan 1;16(6):2798–810. doi: 10.7150/thno.118972 (PMC12775819; doi:10.7150/thno.118972)
Supplement: Supplementary file 1 — Supplementary methods, figures and tables. [file thnov16p2798s1.pdf]

SUPPLEMENTARY INFO

Tailoring PSMA-617-Based Inhibitors through Charged Linker Modifications: Insights into Structure–Activity Relationships

Nicolas M. Geis[1,2,3], Yvonne Braunwarth[1], Phillip T. Meyer[1], Matthias Eder[1,2], Ann-Christin Eder[1,2*]

[1] Department of Nuclear Medicine, University Medical Center Freiburg, Faculty of Medicine, University of Freiburg, Hugstetter Str. 55, 79106 Freiburg, Germany.

[2] Division of Radiopharmaceutical Development, German Cancer Consortium, Partner Site Freiburg, Hugstetter Str. 55, 79106 Freiburg, Germany, and German Cancer Research Center, Im Neuenheimer Feld 280, 69120 Heidelberg, Germany

[3] Faculty of Biology, University of Freiburg, 79104 Freiburg, Germany

E-mail addresses:

nicolas.geis@uniklinik-freiburg.de; yvonne.kiefer@uniklinik-freiburg.de; philipp.meyer@uniklinik-freiburg.de; matthias.eder@uniklinik-freiburg.de; ann-christin.eder@uniklinik-freiburg.de

***Correspondence to:**

Dr. Ann-Christin Eder
Department of Nuclear Medicine,
University Medical Center Freiburg, Faculty of Medicine, University of Freiburg
Hugstetter Str. 55
79106 Freiburg i. Br.
Germany
e-mail: ann-christin.eder@uniklinik-freiburg.de
phone: +49 (0)761 270 74221

Table of contents

S1. General Info

S2. Supplementary Methods

S2. Supplementary Figures

Figure S1: Schematic representation of the synthesis of the PSMA binding motif consisting of glutamic acid, urea and lysine (EuK). 3

Figure S2: Schematic representation of the synthesis of the linker modified PSMA inhibitors PS1 - PS11 starting from the the EuK binding-motif and utilizing solid phase peptide synthesis with a Fmoc-strategy. 3

Figure S3: Chemical structure (A), analytical HPLC (B) and mass spectrometry (C) of PS1 (EuK-HEHEHE-2NaI-ChX-DOTA) according to the methods described in S2. Supplementary Methods. 4

Figure S4: Chemical structure (A), analytical HPLC (B) and mass spectrometry (C) of PS2 (EuK-2NaI-HEHEHE-ChX-DOTA) according to the methods described in S2. Supplementary Methods. 5

Figure S5: Chemical structure (A), analytical HPLC (B) and mass spectrometry (C) of PS3 (EuK-2NaI-ChX-HEHEHE-DOTA) according to the methods described in the S2. Supplementary Methods. 6

Figure S6: Chemical structure (A), analytical HPLC (B) and mass spectrometry (C) of PS4 (EuK-2NaI-ChX-EHEHEH-DOTA) according to the methods described in S2. Supplementary Methods. 7

Figure S7: Chemical structure (A), analytical HPLC (B) and mass spectrometry (C) of PS5 (EuK-2NaI-ChX-HEHEHEHE-DOTA) according to the methods described in S2. Supplementary Methods. 8

Figure S8: Chemical structure (A), analytical HPLC (B) and mass spectrometry (C) of PS6 (EuK-EEE-2NaI-ChX-DOTA) according to the methods described in S2. Supplementary Methods. 9

Figure S9 Chemical structure (A), analytical HPLC (B) and mass spectrometry (C) of PS7 (EuK-2NaI-EEE-ChX-DOTA) according to the methods described in S2. Supplementary Methods. 10

Figure S10: Chemical structure (A), analytical HPLC (B) and mass spectrometry (C) spectrometry of PS8 (EuK-2NaI-ChX-EEE-DOTA) according to the methods described in S2. Supplementary Methods. 11

Figure S11: Chemical structure (A), analytical HPLC (B) and mass spectrometry (C) of PS9 (EuK-HHH-2NaI-ChX-DOTA) according to the methods described in S2. Supplementary Methods. 12

Figure S12: Chemical structure (A), analytical HPLC (B) and mass spectrometry (C) of PS10 (EuK-2NaI-HHH-ChX-DOTA) according to the methods described in S2. Supplementary Methods. 13

Figure S13: Chemical structure (A), analytical HPLC (B) and mass spectrometry (C) of PS11 (EuK-2NaI-ChX-HHH-DOTA) according to the methods described in S2. Supplementary Methods. 14

Figure S14: Analytical radio HPLCs of [^{68}Ga]Ga-PS1 and [^{177}Lu]Lu-PS1. 15

Figure S15: Analytical radio HPLCs of [^{68}Ga]Ga-PS2 and [^{177}Lu]Lu-PS2. 15

Figure S16: Analytical radio HPLCs of [^{68}Ga]Ga-PS3 and [^{177}Lu]Lu-PS3. 16

Figure S17: Analytical radio HPLCs of [^{68}Ga]Ga-PS4 and [^{177}Lu]Lu-PS4. 16

Figure S18: Analytical radio HPLCs of [^{68}Ga]Ga-PS5 and [^{177}Lu]Lu-PS5. 17

Figure S19: Analytical radio HPLCs of [^{68}Ga]Ga-PS6 and [^{177}Lu]Lu-PS6. 17

Figure S20: Analytical radio HPLCs of [^{68}Ga]Ga-PS7 and [^{177}Lu]Lu-PS7. 18

Figure S21: Analytical radio HPLCs of [^{68}Ga]Ga-PS8 and [^{177}Lu]Lu-PS8. 18

Figure S22: Analytical radio HPLCs of [^{68}Ga]Ga-PS9 and [^{177}Lu]Lu-PS9. 19

Figure S23: Analytical radio HPLCs of [^{68}Ga]Ga-PS10 and [^{177}Lu]Lu-PS10. 19

Figure S24: Analytical radio HPLCs of [^{68}Ga]Ga-PS11 and [^{177}Lu]Lu-PS11. 20

Figure S25: Analytical radio HPLCs of [^{68}Ga]Ga-PSMA-617 and [^{177}Lu]Lu-PSMA-617. 20

Figure S26: Time-activity curves (0 - 60 min) of PET scans from ^{68}Ga-labeled compounds showing accumulations in organs of interest. 21

Figure S27: MIPS's from ^{68}Ga-labeled compounds 1 h p.i. of 0.5 nmol compound in LNCaP tumor-bearing BALB/c nu/nu mice. 21

S4. Supplementary Tables

Table S1: p-values of the Partition Coefficient (logD) of the ^{68}Ga-labeled compounds. 22

Table S2: Mouse serum stability of the ^{177}Lu-labeled compounds. 22

Table S3: Mouse serum stability of the ^{177}Lu-labeled compounds. 22

Table S4: p-values of the compounds of the Competetive Cell Binding Assays. 23

Table S5: Surface Binding and Internalization of the ^{68}Ga-labeled compounds. 23

Table S6: p-values of the ^{68}Ga-labeled compounds of the Surface Binding Assay. 24

Table S7: p-values of the ^{68}Ga-labeled compounds of the Internalization Assay. 24

Table S8: SUV values of the ^{68}Ga-labeled compounds 1 h and 2 h p.i. of the relevant organs. 25

S1. General Info

Chemicals: Chemicals (>95% purity; for radiolabeling, highly pure chemicals) and solvents (metal-free nanopure water; HPLC-grade solvents) used for synthesis and analysis were purchased commercially from Sigma-Aldrich (Taufkirchen, Germany), Carl Roth (Karlsruhe), Merck (Darmstadt, Germany), Iris Biotech (Marktredwitz, Germany), CheMatech (Dijon, France) and were used without further purification.

^{68}Gallium (^{68}Ga): ^{68}Gallium (^{68}Ga): The radiometal 68Ga (half-life 69 min; β^+ 89 %; $E\beta^+$ max. 1.9 MeV) used was obtained from a 3.7 GBq ^{68}Ge/^{68}Ga generator (GalliaPharm®) from Eckert & Ziegler (Berlin, Germany) in the form of [^{68}Ga]Ga^{3+} eluate ions in acidic solution.

^{177}Lutetium (^{177}Lu): The radiometal ^{177}Lu (half-life 6.65 days; β^- 79 %; 0.50 MeV) was purchased in form of a [^{177}Lu]$LuCl_3$ solution from ITM (Kampen, Netherlands).

S2. Supplementary Methods

Synthesis of the EuK binding motif: 2-Chlorotrityl resin (2-CT resin, 5.00 g, 5.70 mmol, 1.0 eq) was weighed into a dry round bottom flask and allowed to swell in dried dichloromethane (DCM; 100 ml) for 45 min at room temperature. Fmoc-Lys(Alloc)-OH (3.10 g, 6.84 mmol, 1.2 eq) was dissolved in DCM (50 ml) and added slowly to the swollen 2-CT resin alongside *N,N*-diisoprpylethylamine (DIPEA, 4.68 ml, 3.54 g, 27.4 mmol, 4.8 eq) and shaken slowly over 16 h at room temperature.

The DCM was removed the next day and the residue was washed 6× with DCM and unreacted chlorotrityl groups were blocked with a mixture of DCM/methanol/DIPEA in a ratio of 17:2:1 and the residue was washed a further 6× with DCM.

To remove the Fmoc protecting group, the lysine-coupled resin (ca. 350 mg, 0.3 eq) was allowed to swell in DCM (5 ml) for 30 min and rinsed 6× with dimethylformamide (DMF). The resin was then gently shaken for 2 min and 5 min in a 1:1 mixture of DMF and piperidine and then washed 6× with DMF and 6× with DCM.

To form the isocyanate group on glutamate, triphosgene (296.8 mg, 1.00 mmol, 1.0 eq) was first weighed into a round-necked flask and dissolved in DCM (10 ml). The bis(*t*Bu)-L-glutamate (890.4 mg, 3 mmol, 3 eq, 13) was dissolved in DIPEA (2.00 ml, 1.51 g, 11.7 mmol, 11.7 eq) and DCM (200 ml). This solution was slowly added to the dissolved phosgene over 4 h at 0 °C in an ice bath.

After the addition was complete, the deprotected lysine-coupled resin was slowly added dropwise to the triphosgene mixture and then stirred for 16 h at room temperature.

In a next step, the 2-CT resin was separated via a 5 ml syringe with a frit. To remove the Alloc protecting group, tetrakis(triphenylphosphine)-palladium(0) (100 mg, 0.1 mmol, 0.3 eq) was dissolved in DCM (2.7 ml) and morpholine (300 µl, 0.30 g, 3.44 mmol, 11 eq) was added. The mixture was added to the

protected product on the resin in the syringe and slowly rotated for 1 h under exclusion of light at room temperature. The procedure was carried out analogously a second time with fresh tetrakis(triphenylphosphine)-palladium(0) and morpholine and again allowed to rotate slowly for 1 h. The eluate was removed and the mixture was removed from the syringe and washed 6× with DCM and 6× with DMF.
The residue was further rinsed 8× with DIPEA (1 vol% in DMF, 5 ml each) and 8× with sodium diethyl dithiocarbamate trihydrate (90 mM in DMF, 5 ml each) and washed 6× with DCM and 6× with DMF. The residue was dried over diethyl ether and aliquoted in 3 parts of 0.1 mmol each. Representation of the synthesis steps is displayed in Sup. Fig. 1.
Further synthesis steps were carried out utilizing solid phase synthesis via Fmoc strategy (Figure S2).

Analytical HPLC: Analytical high performance liquid chromatography was carried out using an Agilent 1100 series system (Agilent Technologies, USA) equipped with a dual UV detector (1200 Series DAD; monitored wavelength: 220/280 nm; Agilent Technologies, USA) and a Chromolith RP-18e (100 × 4.6 mm, 2 µm, 130Å; Merck, USA) column. A linear gradient (95 % A (0.1 % TFA in H_2O) to 95 % B (0.1 % TFA in CH_3CN)) in 10 min at 1 mL/min was used.

Analytical radio HPLC: Analytical radio high performance liquid chromatography was carried out using an Agilent 1100 series system (Agilent Technologies, USA) equipped with a gamma counter (RAMONA; Elysia-Raytest, Straubenhardt, Germany) and a Chromolith RP-18e (100 × 4.6 mm, 2 µm, 130Å; Merck, USA) column. A linear gradient (95 % A (0.1 % TFA in H_2O) to 95 % B (0.1 % TFA in CH_3CN)) in 14 min at 1 mL/min was used.

Thin-Layer Chromatography (TLC): Radiochemical purity of the radiolabeled PSMA compounds was assessed using thin-layer chromatography (TLC) on cellulose-based strips. The mobile phase consisted of a 1:1 mixture of 10 % ammonium acetate (NH_4OAc) and dimethylformamide. The strips were analyzed using a radio-TLC scanner.

Purification: Purifications were performed utilizing the automated reverse phase flash chromatography Isolera Prime System (Biotage, Sweden), equipped with a Sfär Bio C18 column (Duo 300 Å, 20 µm; Biotage, Sweden). Different gradients (70 - 95 % A (0.1 % TFA in H_2O) to 95 % B (0.1 % TFA in CH_3CN)) in 10 - 30 min at 25 ml/min were used.

Mass spectrometry: For mass spectrometry, a Flexar SQ 300 MS ESI system (PerkinElmer, USA) was used. Tracer amounts of substance were dissolved in ultrapure water and directly injected into the ESI.

S3. Supplementary Figures

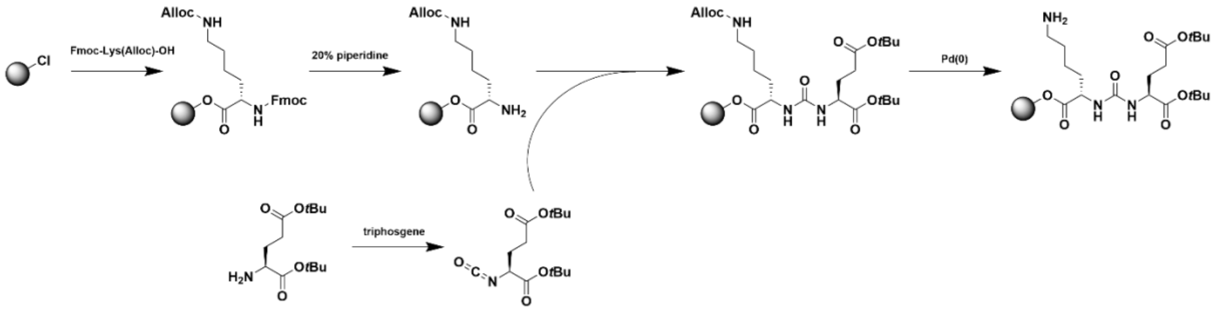

Figure S1: Schematic representation of the synthesis of the PSMA binding motif consisting of glutamic acid, urea and lysine (EuK).

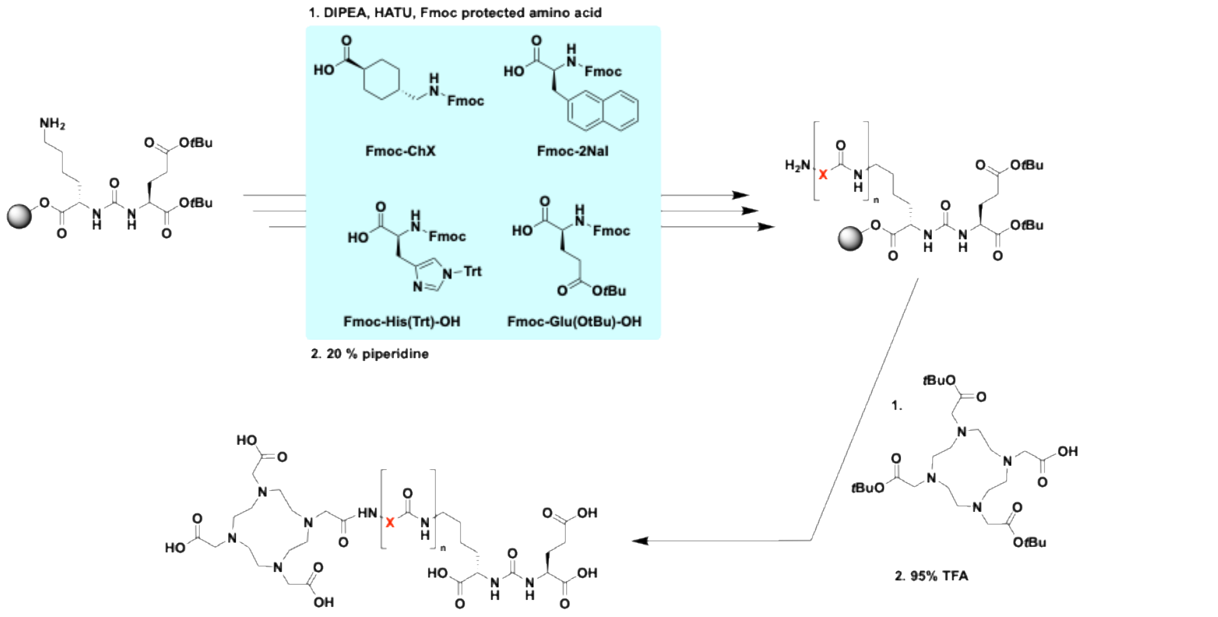

Figure S2: Schematic representation of the synthesis of the linker modified PSMA inhibitors PS1 - PS11 starting from the the EuK binding-motif and utilizing solid phase peptide synthesis with a Fmoc-strategy.

PS1 (EuK-**HEHEHE**-2NaI-ChX-DOTA)

A

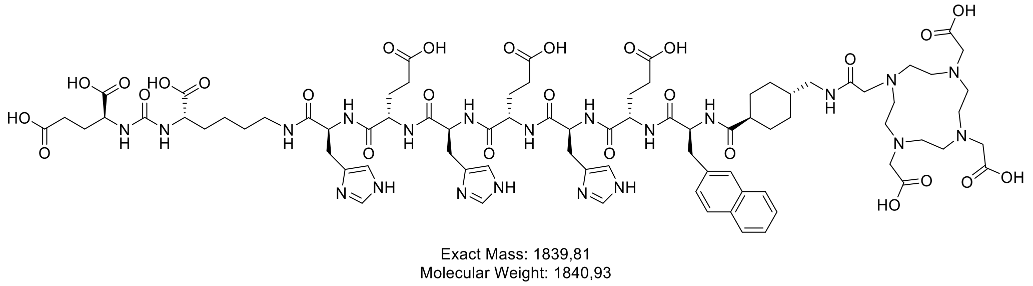

B

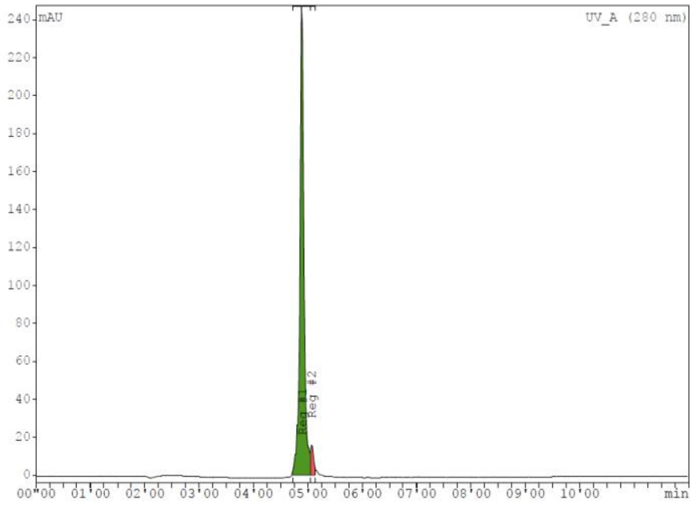

Integration UV_A (280 nm)

Substanz	Ret s	Typ	Fläche mAU*s	%Fläche %
Reg #1	04'54	DD(M	1268,270	95,45
Reg #2	05'05	DD(M	60,460	4,55
Summe in ROI			1328,730	

C

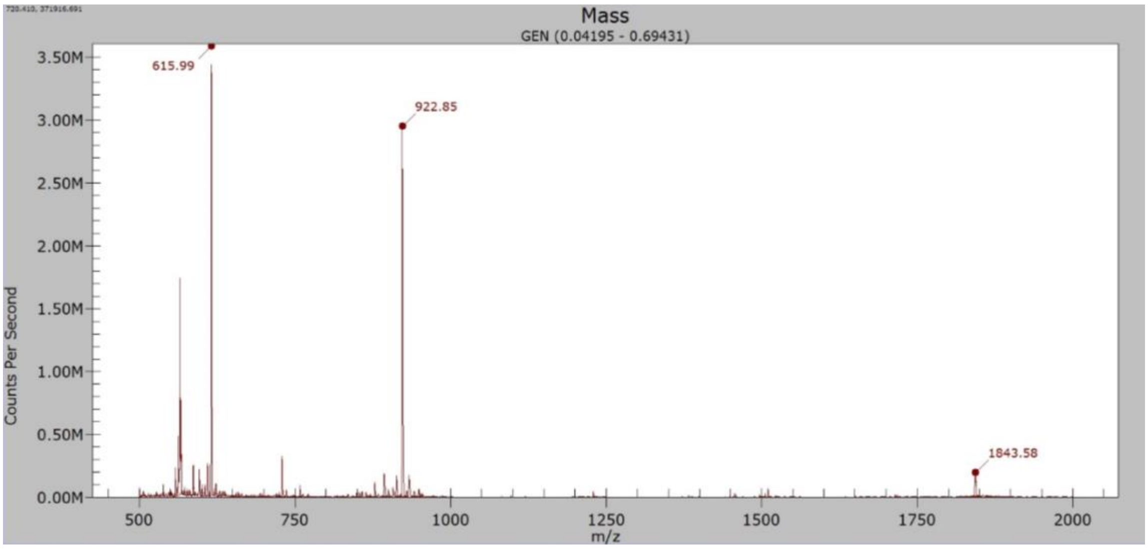

Figure S3: Chemical structure (A), analytical HPLC (B) and mass spectrometry (C) of PS1 (EuK-**HEHEHE**-2NaI-ChX-DOTA) according to the methods described in S2. Supplementary Methods.

PS2 (EuK-2NaI-**HEHEHE**-ChX-DOTA)

A

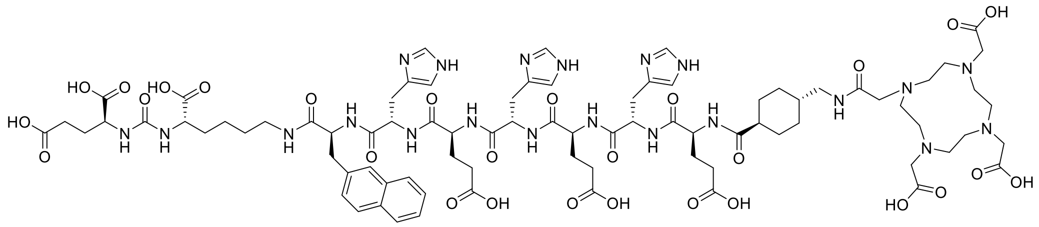

Molecular Weight: 1840,93

B

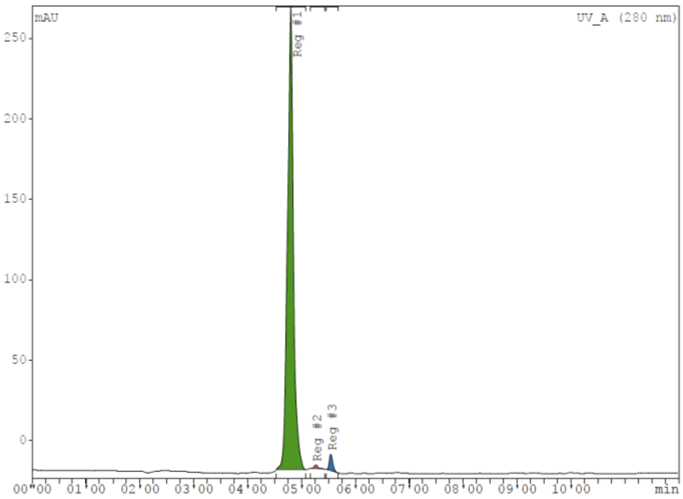

Integration UV_A (280 nm)

Substanz	Ret s	Typ	Fläche mAU*s	%Fläche %
Reg #1	04'48	BB(M	2181,501	97,47
Reg #2	05'16	BB(M	11,051	0,49
Reg #3	05'33	BB(M	45,676	2,04
Summe in ROI			2238,228	

C

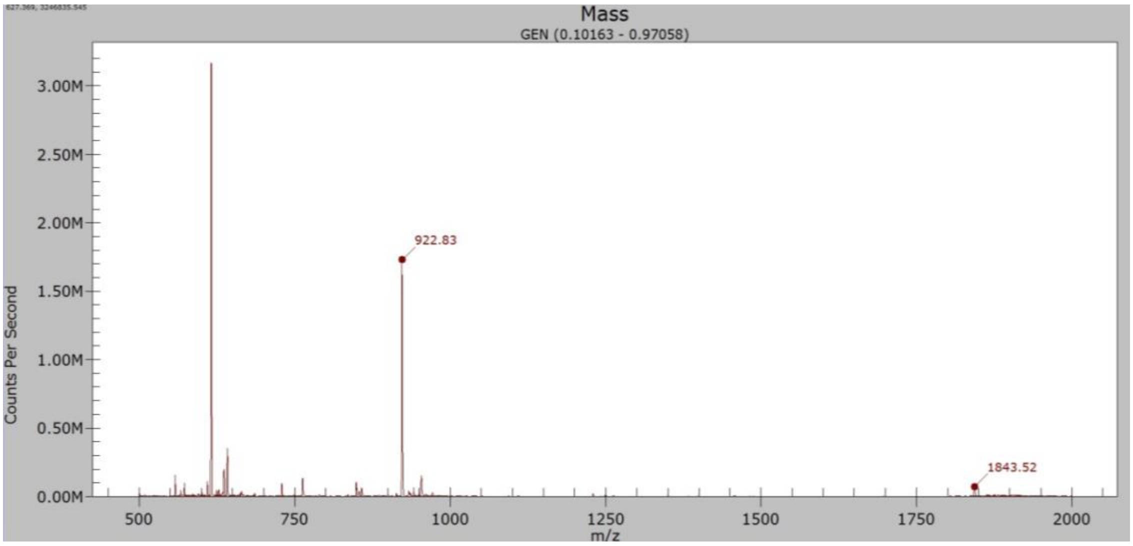

Figure S4: Chemical structure (A), analytical HPLC (B) and mass spectrometry (C) of PS2 (EuK-2NaI-**HEHEHE**-ChX-DOTA) according to the methods described in S2. Supplementary Methods.

PS3 (EuK-2NaI-ChX-**HEHEHE**-DOTA)

A

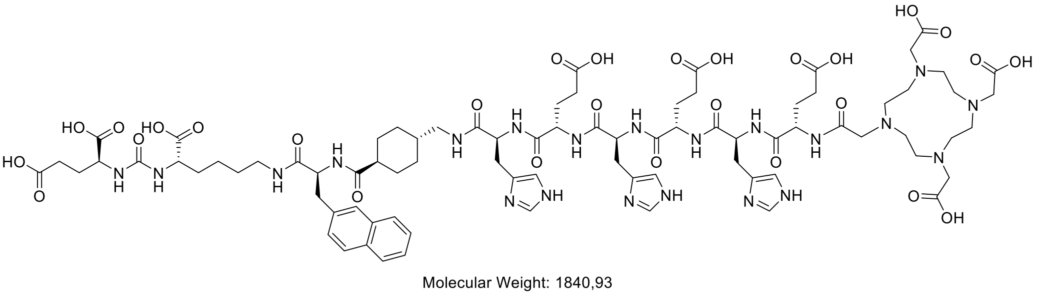

B

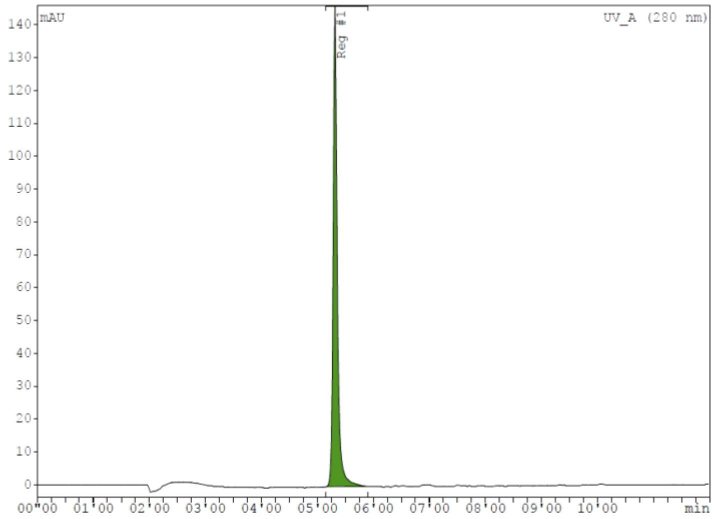

Integration UV_A (280 nm)

Substanz	Ret s	Typ	Fläche mAU*s	%Fläche %
Reg #1	05'19	BB(M	750,5672	100,00
Summe in ROI			750,5672	

C

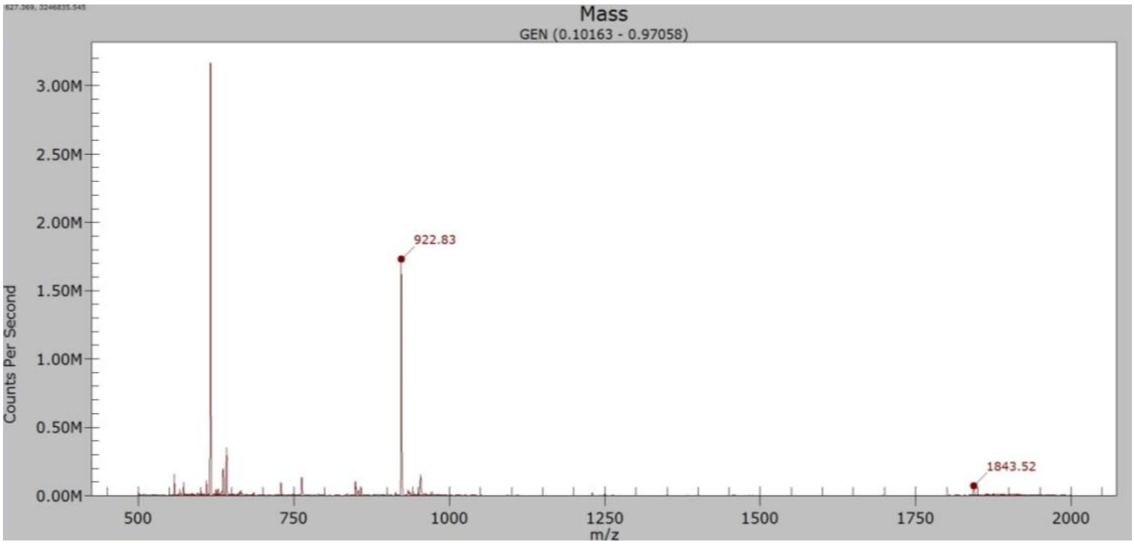

Figure S5: Chemical structure (A), analytical HPLC (B) and mass spectrometry (C) of PS3 (EuK-2NaI-ChX-**HEHEHE**-DOTA) according to the methods described in the S2. Supplementary Methods.

PS4 (EuK-2NaI-ChX-**EHEHEH**-DOTA)

A

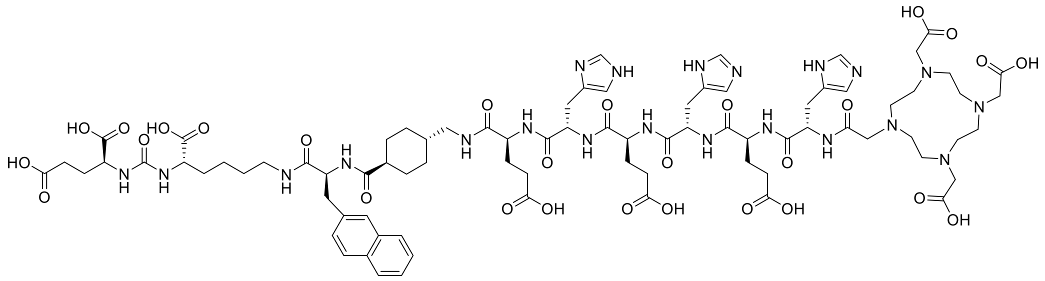

Molecular Weight: 1840,93

B

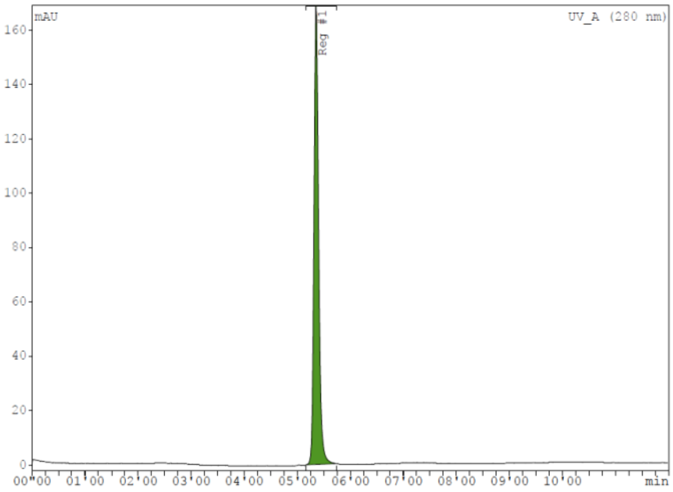

Integration UV_A (280 nm)

Substanz	Ret s	Typ	Fläche mAU*s	%Fläche %
Reg #1	05'22	BB(M	1019,780	100,00
Summe in ROI			1019,780	

C

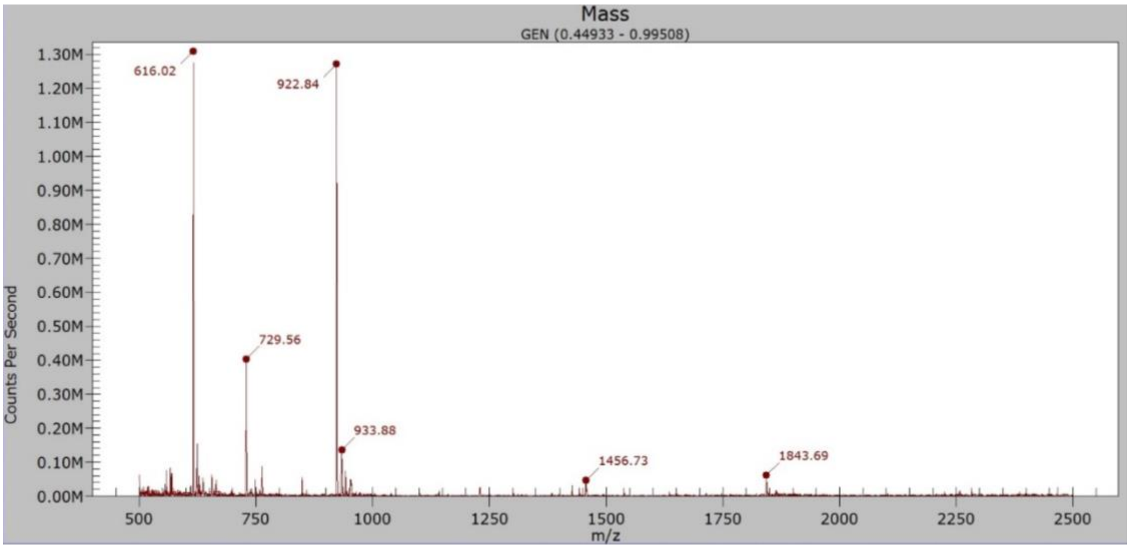

Figure S6: Chemical structure (A), analytical HPLC (B) and mass spectrometry (C) of PS4 (EuK-2NaI-ChX-**EHEHEH**-DOTA) according to the methods described in S2. Supplementary Methods.

PS5 (EuK-2NaI-ChX-**HEHEHEHE**-DOTA)

A

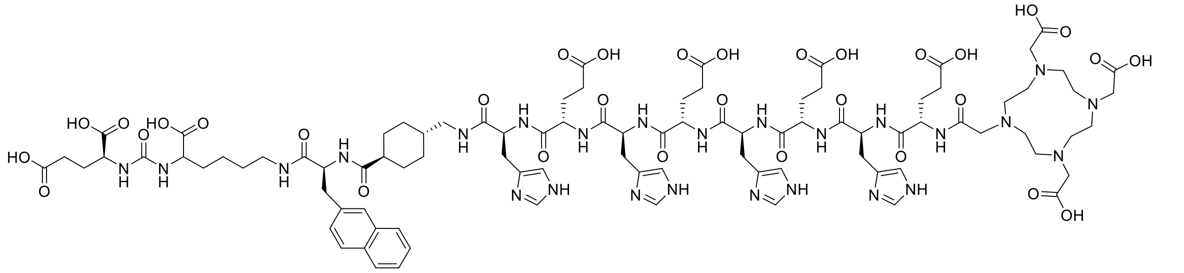

Molecular Weight: 2107,18

B

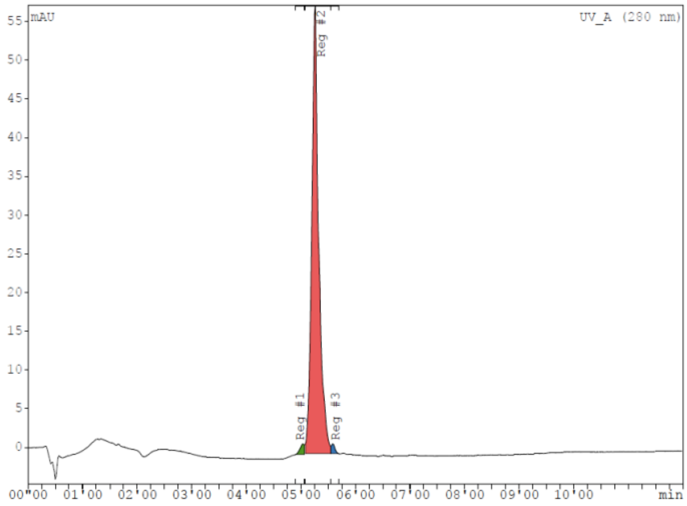

Integration UV_A (280 nm)

Substanz	Ret s	Typ	Fläche mAU*s	%Fläche %
Reg #1	04'58	BD(M	6,6128	1,27
Reg #2	05'15	DD(M	508,9305	97,54
Reg #3	05'37	DB(M	6,2146	1,19
Summe in ROI			521,7579	

C

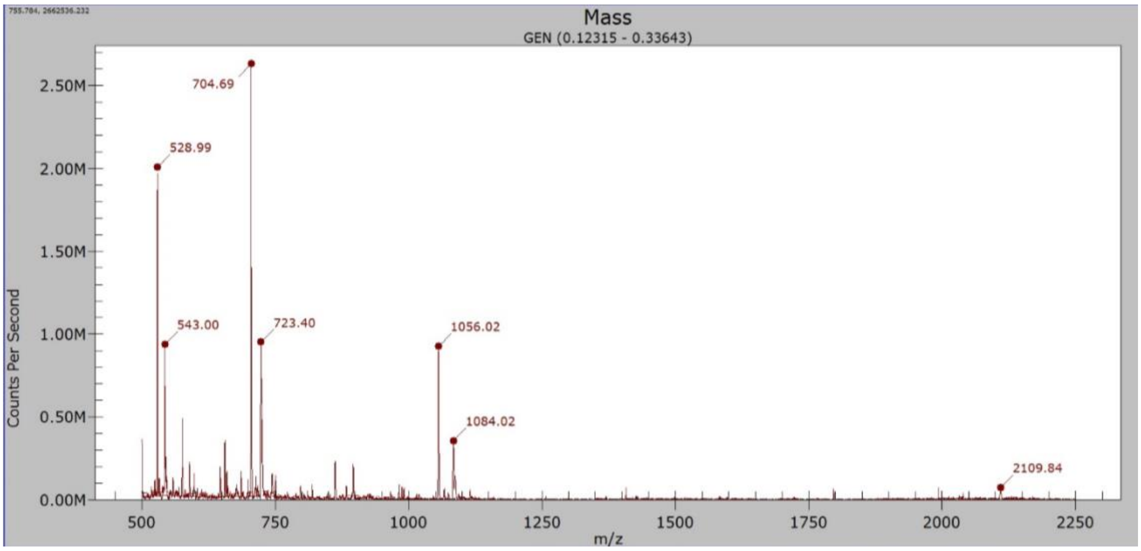

Figure S7: Chemical structure (A), analytical HPLC (B) and mass spectrometry (C) of PS5 (EuK-2NaI-ChX-**HEHEHEHE**-DOTA) according to the methods described in S2. Supplementary Methods.

PS6 (EuK-**EEE**-2NaI-ChX-DOTA)

A

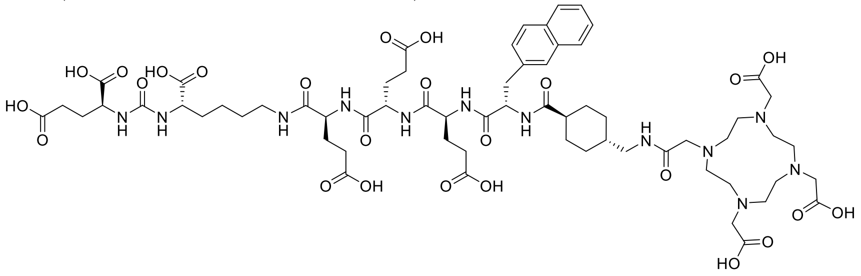

Molecular Weight: 1429,50

B

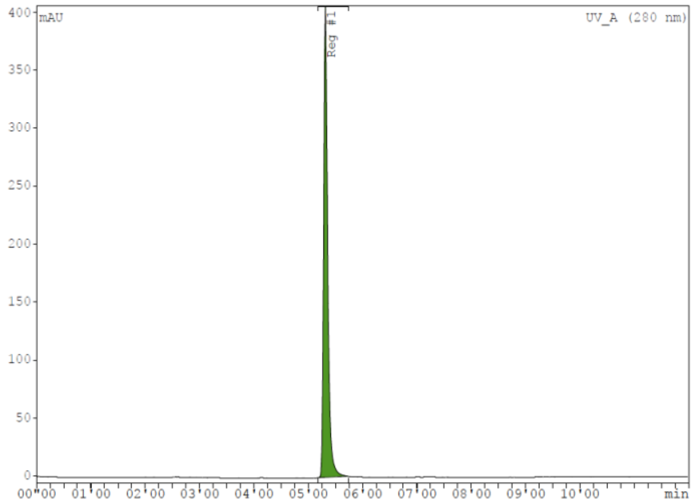

Integration UV_A (280 nm)

Substanz	Ret s	Typ	Fläche mAU*s	%Fläche %
Reg #1	05'19	BB(M	2093,906	100,00
Summe in ROI			2093,906	

C

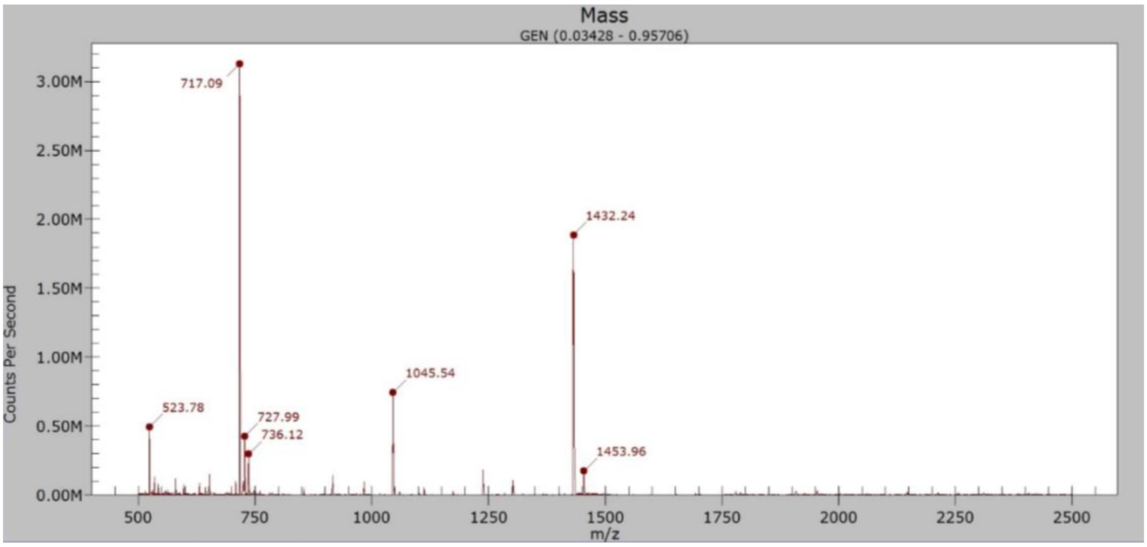

Figure S8: Chemical structure (A), analytical HPLC (B) and mass spectrometry (C) of PS6 (EuK-**EEE**-2NaI-ChX-DOTA) according to the methods described in S2. Supplementary Methods.

PS7 (EuK-2NaI-**EEE**-ChX-DOTA)

A

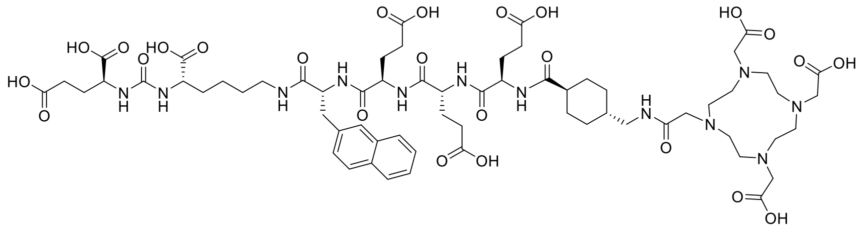

Molecular Weight: 1429,50

B

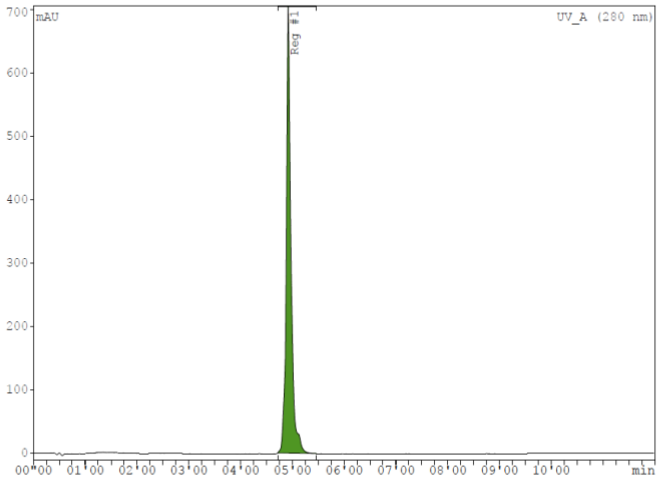

Integration UV_A (280 nm)

Substanz	Ret s	Typ	Fläche mAU*s	%Fläche %
Reg #1	04'56	BB(M	4376,473	100,00
Summe in ROI			4376,473	

C

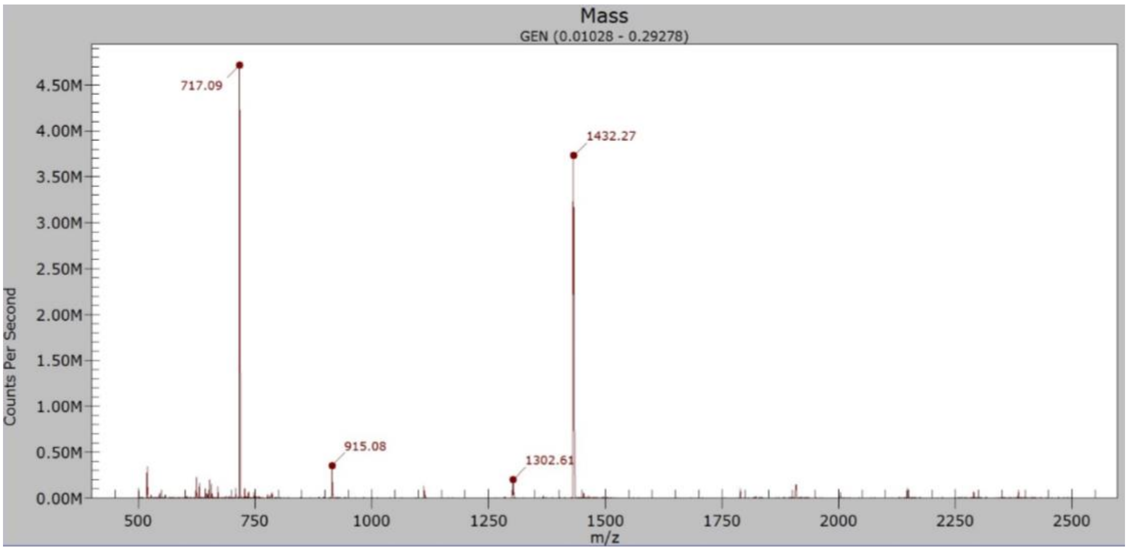

Figure S9 Chemical structure (A), analytical HPLC (B) and mass spectrometry (C) of PS7 (EuK-2NaI-**EEE**-ChX-DOTA) according to the methods described in S2. Supplementary Methods.

PS8 (EuK-2NaI-ChX-**EEE**-DOTA)

A

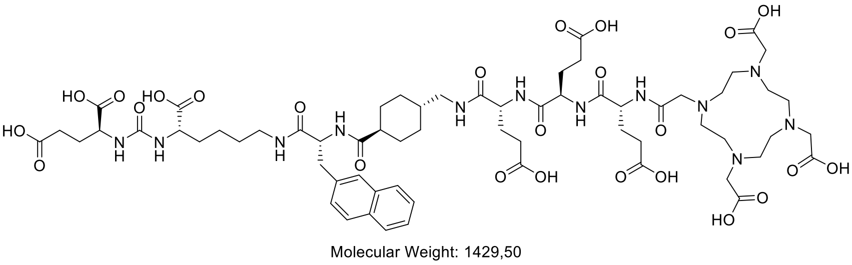

B

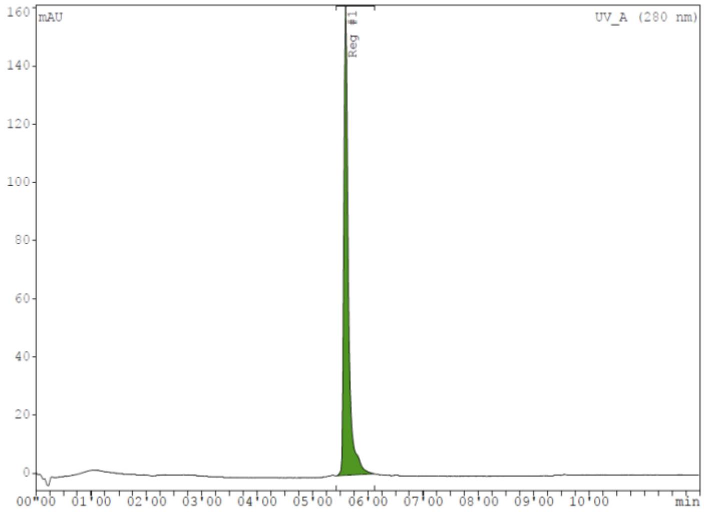

Integration UV_A (280 nm)

Substanz	Ret s	Typ	Fläche mAU*s	%Fläche %
Reg #1	05'36	BB(M	920,8553	100,00
Summe in ROI			920,8553	

C

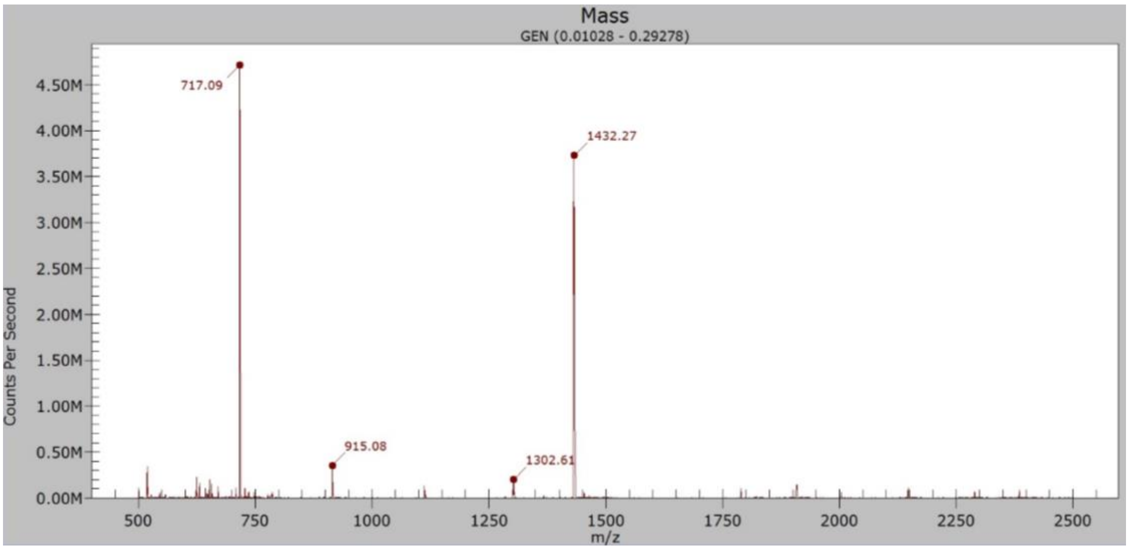

Figure S10: Chemical structure (A), analytical HPLC (B) and mass spectrometry (C) spectrometry of PS8 (EuK-2NaI-ChX-**EEE**-DOTA) according to the methods described in S2. Supplementary Methods.

PS9 (EuK-**HHH**-2NaI-ChX-DOTA)

A

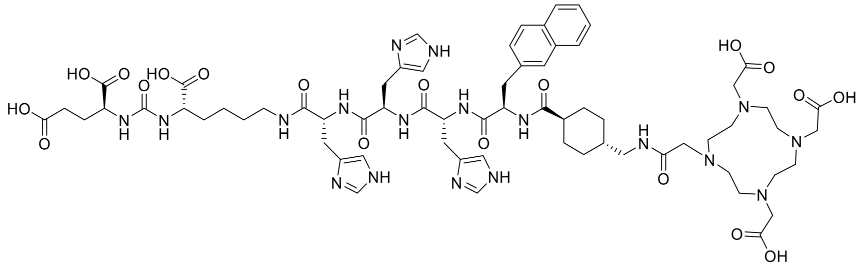

Molecular Weight: 1453,58

B

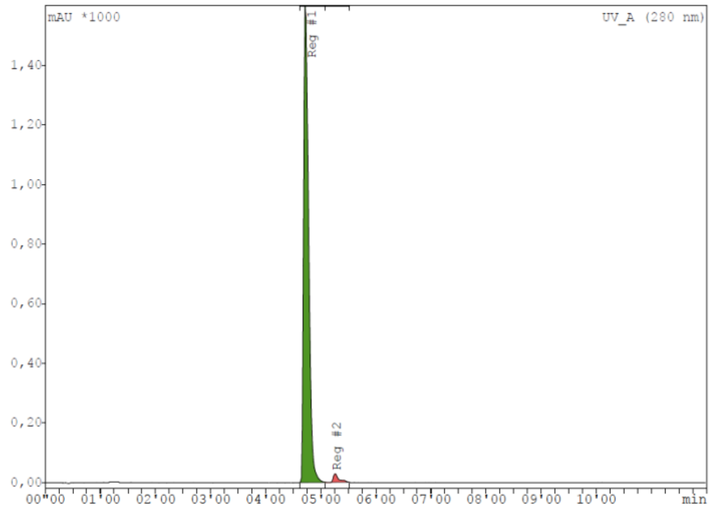

Integration UV_A (280 nm)

Substanz	Ret s	Typ	Fläche mAU*s	%Fläche %
Reg #1	04'44	BD(M	10077,90	98,19
Reg #2	05'16	DB(M	186,05	1,81
Summe in ROI			10263,94	

C

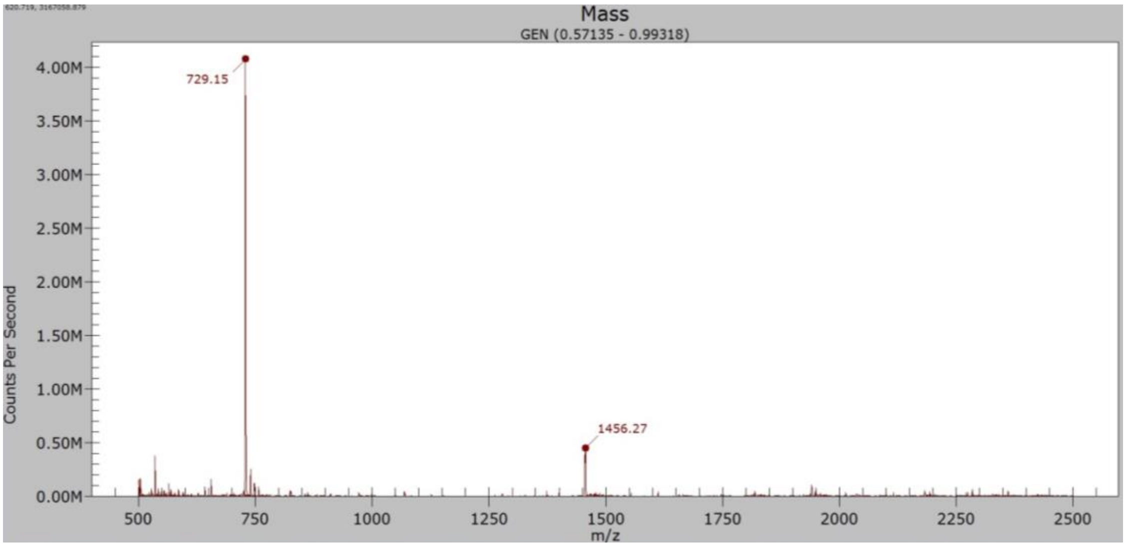

Figure S11: Chemical structure (A), analytical HPLC (B) and mass spectrometry (C) of PS9 (EuK-**HHH**-2NaI-ChX-DOTA) according to the methods described in S2. Supplementary Methods.

PS10 (EuK-2NaI-**HHH**-ChX-DOTA)

A

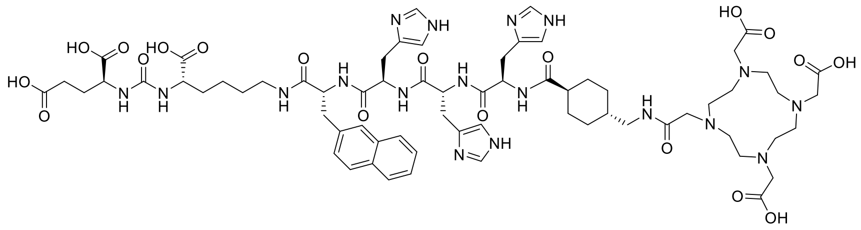

Molecular Weight: 1453,58

B

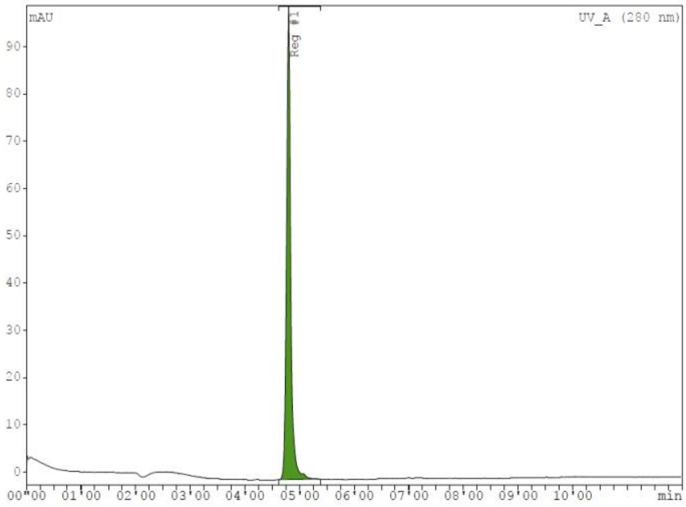

Integration UV_A (280 nm)

Substanz	Ret s	Typ	Fläche mAU*s	%Fläche %
Reg #1	04'48	BB(M	523,7071	100,00
Summe in ROI			523,7071	

C

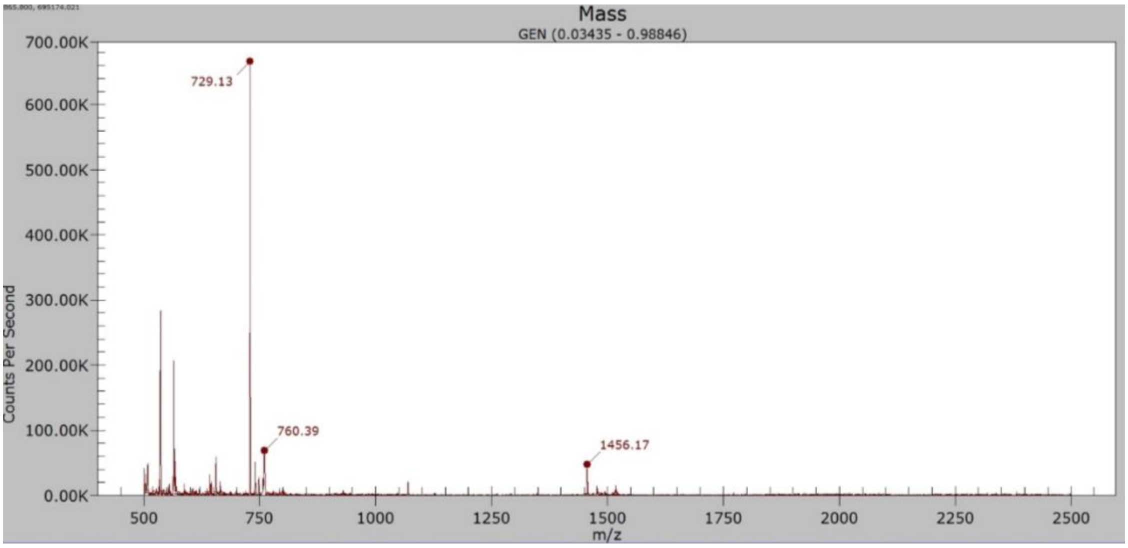

Figure S12: Chemical structure (A), analytical HPLC (B) and mass spectrometry (C) of PS10 (EuK-2NaI-**HHH**-ChX-DOTA) according to the methods described in S2. Supplementary Methods.

PS11 (EuK-2NaI-ChX-**HHH**-DOTA)

A

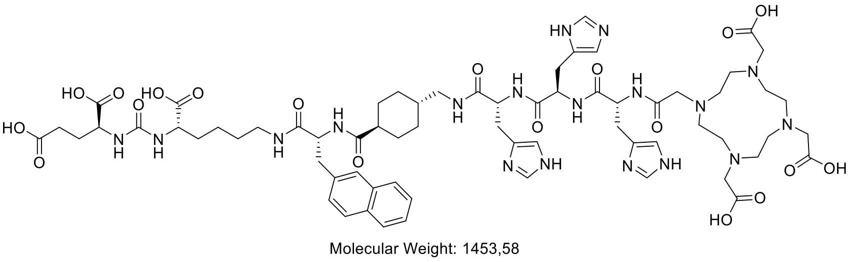

B

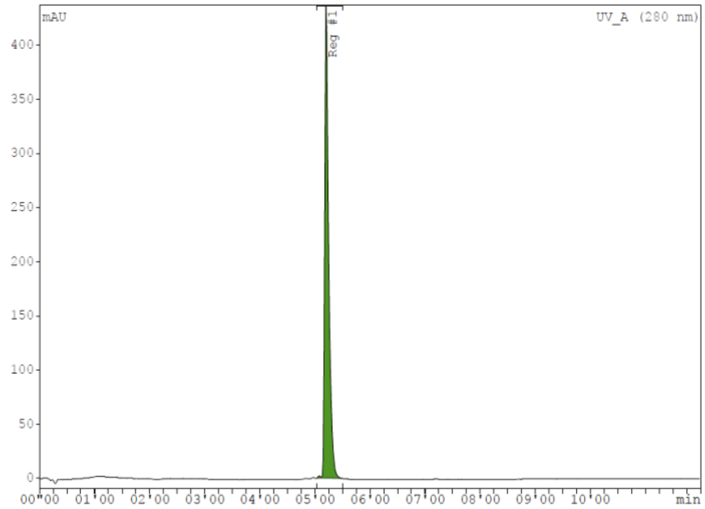

Integration UV_A (280 nm)

Substanz	Ret s	Typ	Fläche mAU*s	%Fläche %
Reg #1	05'13	BB(M	2192,076	100,00
Summe in ROI			2192,076	

C

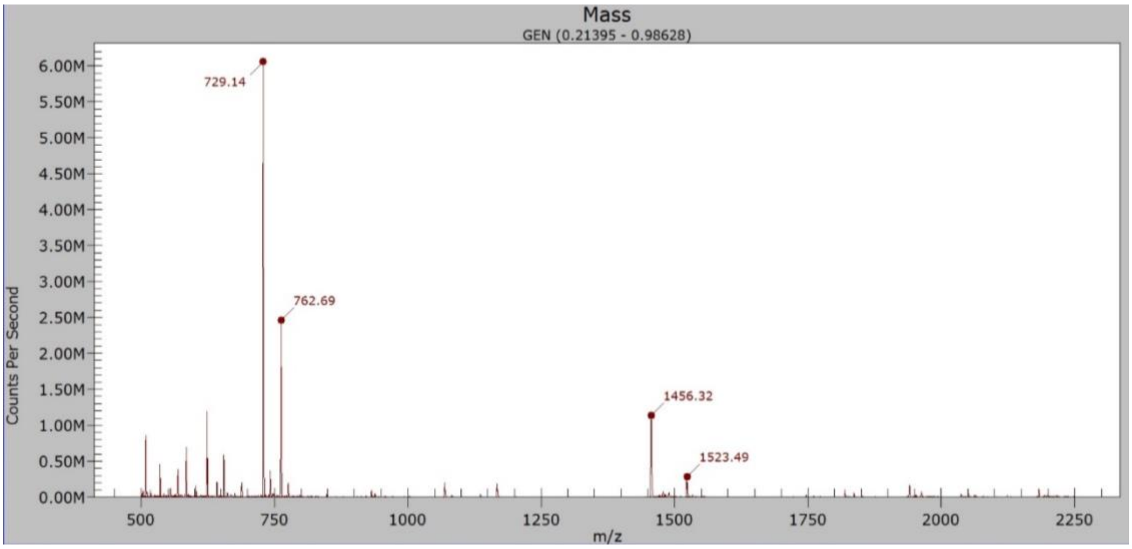

Figure S13: Chemical structure (A), analytical HPLC (B) and mass spectrometry (C) of PS11 (EuK-2NaI-ChX-**HHH**-DOTA) according to the methods described in S2. Supplementary Methods.

Supp. Fig. 14

PS – 1 (EuK-HEHEHE-2Nal-ChX-DOTA)

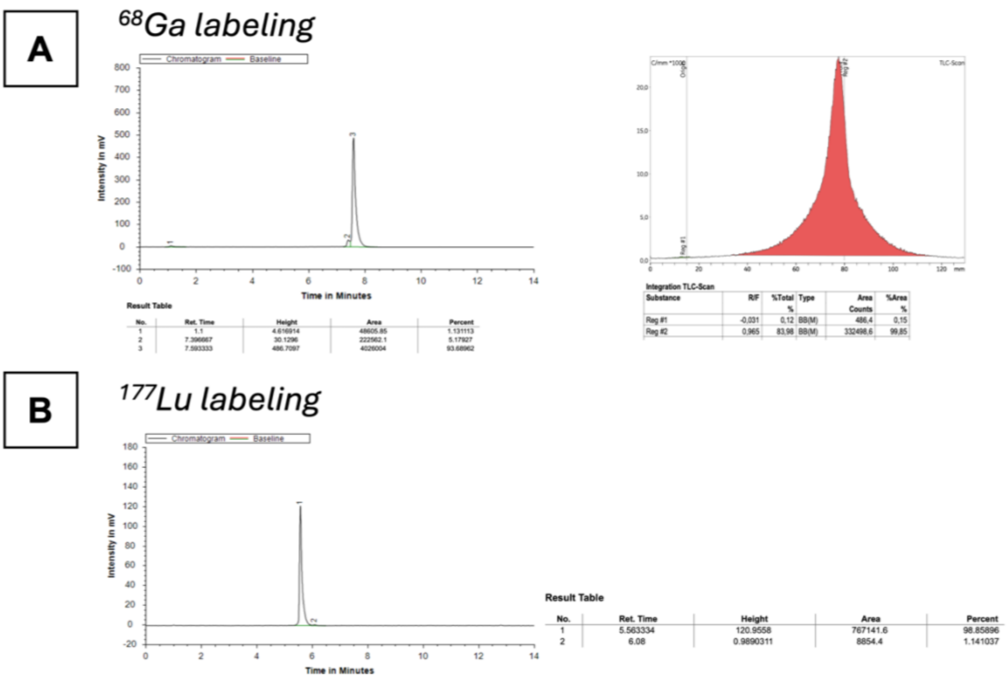

Figure S14: **A**: Analytical radio HPLC and TLC of [^{68}Ga]Ga-PS1; **B**: Analytical radio HPLC [^{177}Lu]Lu-PS1.

Supp. Fig. 15

PS – 2 (EuK-2Nal-HEHEHE-ChX-DOTA)

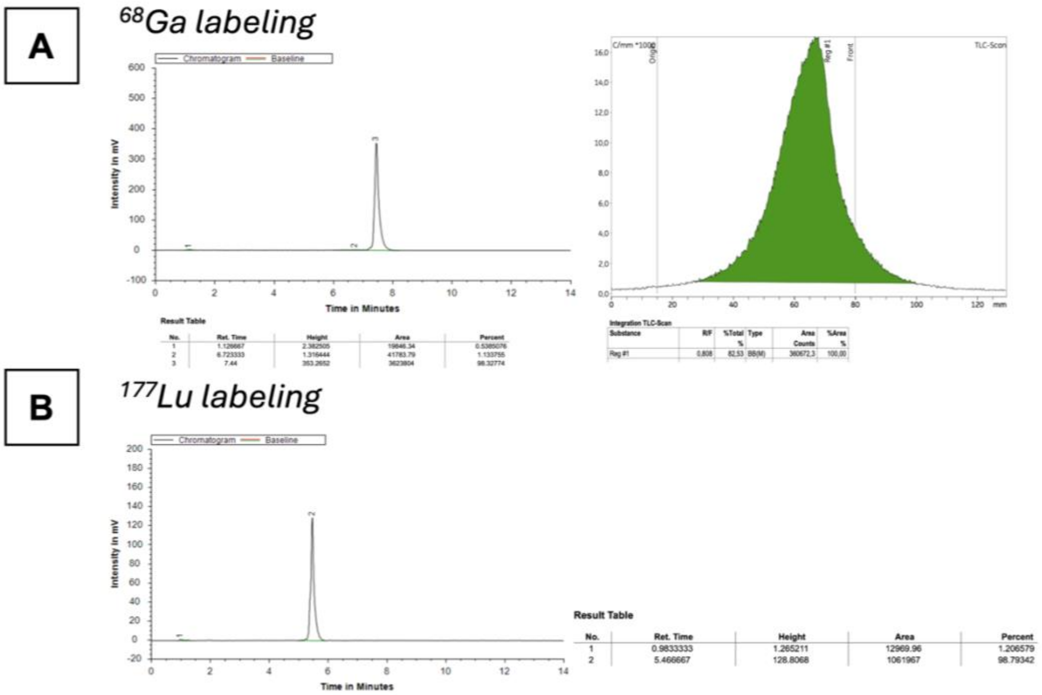

Figure S15: **A**: Analytical radio HPLC and TLC of [^{68}Ga]Ga-PS2; **B**: Analytical radio HPLC [^{177}Lu]Lu-PS2.

Supp. Fig. 16

PS – 3 (EuK-2Nal-ChX-HEHEHE-DOTA)

A

^{68}Ga labeling

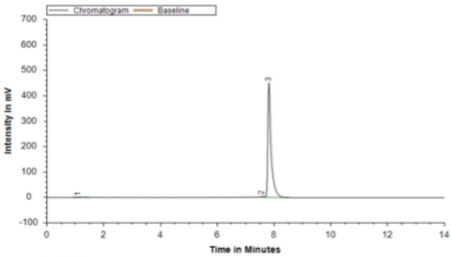

Result Table

No.	Ret. Time	Height	Area	Percent
1	1.106667	1.401121	18543.96	0.50439
2	7.576667	3.798223	26856.77	0.7304958
3	7.823333	450.4861	3631112	98.76511

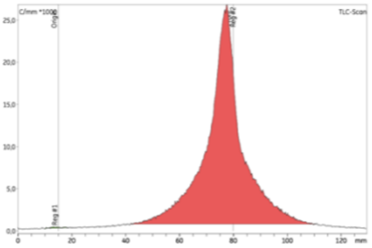

Integration TLC-Scan

Substance	R/F	%Total %	Type	Area Counts	%Area %
Reg #1	-0,027	0,09	BB(M)	396,4	0,11
Reg #2	0,962	81,41	BB(M)	364413,9	99,89

B

^{177}Lu labeling

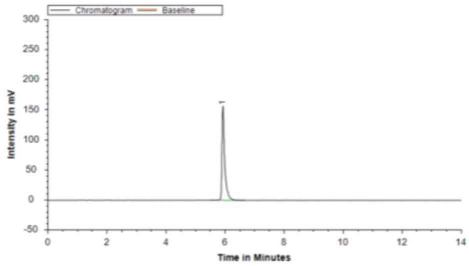

Result Table

No.	Ret. Time	Height	Area	Percent
1	5.926667	156.6344	1009165	100

Figure S16: **A**: Analytical radio HPLC and TLC of [^{68}Ga]Ga-PS3; **B**: Analytical radio HPLC [^{177}Lu]Lu-PS3.

Supp. Fig. 17

PS – 4 (EuK-2Nal-ChX-EHEHEH-DOTA)

A

^{68}Ga labeling

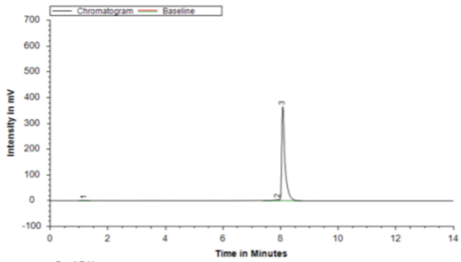

Result Table

No.	Ret. Time	Height	Area	Percent
1	1.183333	0.9517302	9917.516	0.3378052
2	7.906667	4.73063	41281.63	1.406113
3	8.073334	364.7326	2884669	98.25608

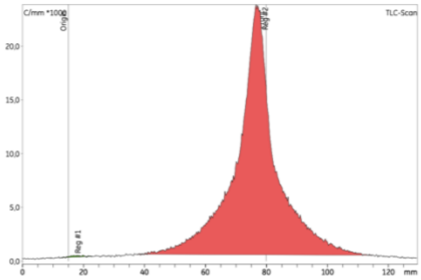

Integration TLC-Scan

Substance	R/F	%Total %	Type	Area Counts	%Area %
Reg #1	0,042	0,14	BB(M)	547,7	0,16
Reg #2	0,954	83,36	BB(M)	333932,0	99,84

B

^{177}Lu labeling

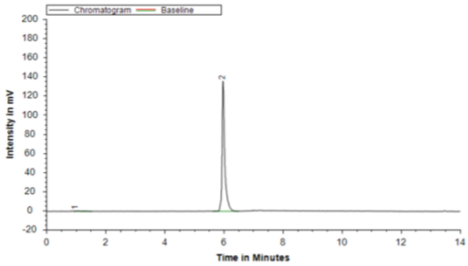

Result Table

No.	Ret. Time	Height	Area	Percent
1	0.9733334	0.4286274	5701.809	0.6634736
2	5.96	135.6029	853685.6	99.33652

Figure S17: **A**: Analytical radio HPLC and TLC of [^{68}Ga]Ga-PS4; **B**: Analytical radio HPLC [^{177}Lu]Lu-PS4.

Supp. Fig. 18

PS – 5 (EuK-2Nal-ChX-HEHEHEHE-DOTA)

A

^{68}Ga labeling

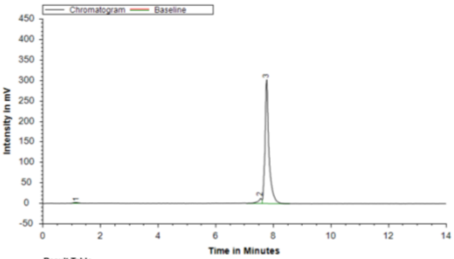

Result Table

No.	Ret. Time	Height	Area	Percent
1	1.166667	1.952078	24852.71	0.8294735
2	7.55	12.40633	115066.7	3.840418
3	7.766667	301.7329	2856283	95.33011

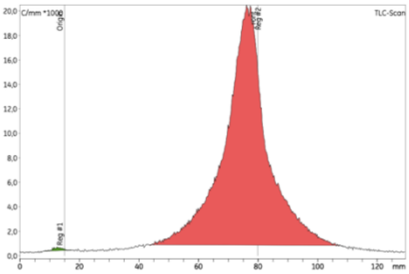

Integration TLC-Scan

Substance	R/F	%Total %	Type	Area Counts	%Area %
Reg #1	-0,031	0,22	BB(M)	890,7	0,28
Reg #2	0,942	78,34	BB(M)	316836,0	99,72

B

^{177}Lu labeling

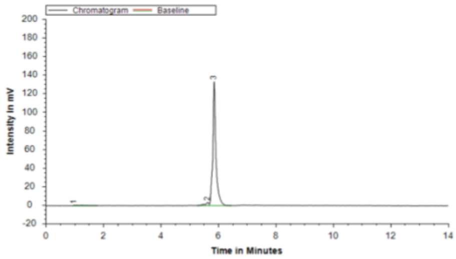

Result Table

No.	Ret. Time	Height	Area	Percent
1	0.9766667	0.7783146	13965.59	1.31243
2	5.63	3.459167	33367.72	3.135765
3	5.846667	133.1662	1016768	95.5518

Figure S18: **A**: Analytical radio HPLC and TLC of [^{68}Ga]Ga-PS5; **B**: Analytical radio HPLC [^{177}Lu]Lu-PS5.

Supp. Fig. 19

PS – 6 (EuK-EEE-2Nal-ChX-DOTA)

A

^{68}Ga labeling

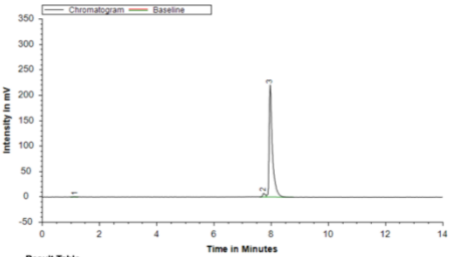

Result Table

No.	Ret. Time	Height	Area	Percent
1	1.15	0.444227	2248.022	0.1271498
2	7.733333	6.893783	43687.3	2.470986
3	7.963333	220.2608	1722076	97.40186

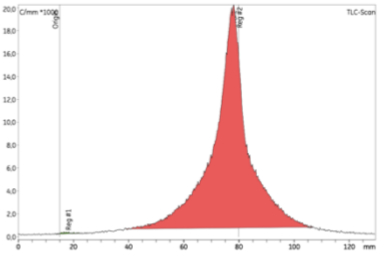

Integration TLC-Scan

Substance	R/F	%Total %	Type	Area Counts	%Area %
Reg #1	0,042	0,12	BB(M)	415,0	0,15
Reg #2	0,969	78,16	BB(M)	268280,1	99,85

B

^{177}Lu labeling

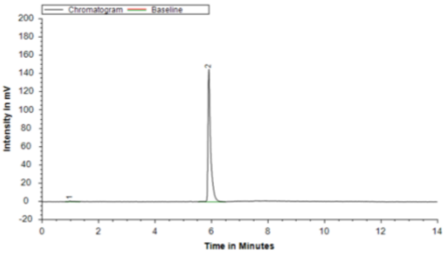

Result Table

No.	Ret. Time	Height	Area	Percent
1	0.9866667	0.7830563	6408.319	0.671109
2	5.903333	144.9466	948476.8	99.32889

Figure S19: **A**: Analytical radio HPLC and TLC of [^{68}Ga]Ga-PS6; **B**: Analytical radio HPLC [^{177}Lu]Lu-PS6.

Supp. Fig. 20

PS – 7 (EuK-2Nal-EEE-ChX-DOTA)

A

^{68}Ga labeling

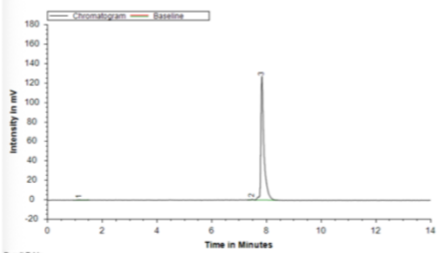

Result Table

No.	Ret. Time	Height	Area	Percent
1	1.16	0.5307342	2873.548	0.2869736
2	7.473333	0.6393813	1547.002	0.154495
3	7.83	126.8867	996907.8	99.55853

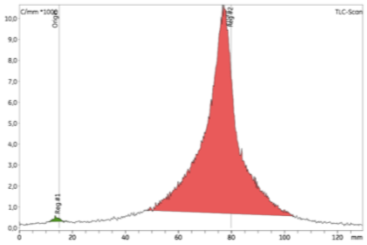

Integration TLC-Scan

Substance	R/F	%Total %	Type	Area Counts	%Area %
Reg #1	-0,015	0,30	BB(M)	604,0	0,44
Reg #2	0,958	68,02	BB(M)	135996,4	99,56

B

^{177}Lu labeling

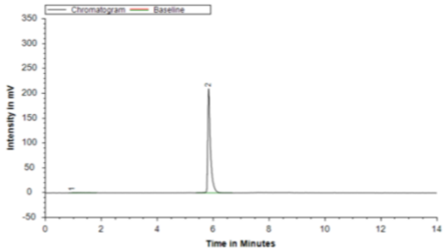

Result Table

No.	Ret. Time	Height	Area	Percent
1	1.16	0.5307342	2873.548	0.2869736
2	7.473333	0.6393813	1547.002	0.154495
3	7.83	126.8867	996907.8	99.55853

Figure S20: **A**: Analytical radio HPLC and TLC of [^{68}Ga]Ga-PS7; **B**: Analytical radio HPLC [^{177}Lu]Lu-PS7.

Supp. Fig. 21

PS – 8 (EuK-2Nal-ChX-EEE-DOTA)

A

^{68}Ga labeling

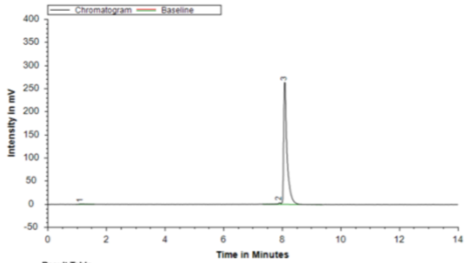

Result Table

No.	Ret. Time	Height	Area	Percent
1	1.11	0.8438738	5643.749	0.2664766
2	7.893333	3.165973	23146.05	1.09287
3	8.076667	263.722	2089126	98.64066

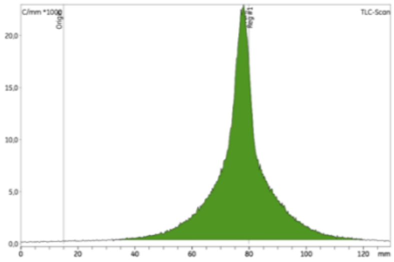

Integration TLC-Scan

Substance	R/F	%Total %	Type	Area Counts	%Area %
Reg #1	0,965	87,50	BB(M)	306847,1	100,00

B

^{177}Lu labeling

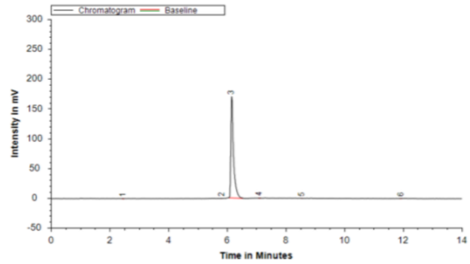

Result Table

No.	Ret. Time	Height	Area	Percent
1	2.453333	0.2240188	719.5192	0.06699289
2	5.82	0.3094393	708.3306	0.06595114
3	6.146667	169.9242	1070618	99.68298
4	7.093333	0.4156411	646.0679	0.060154
5	8.553333	0.2126723	639.2897	0.0595229
6	11.93667	0.2663003	691.6216	0.06439541

Figure S21: **A**: Analytical radio HPLC and TLC of [^{68}Ga]Ga-PS8; **B**: Analytical radio HPLC [^{177}Lu]Lu-PS8.

Supp. Fig. 22

PS – 9 (EuK-HHH-2Nal-ChX-DOTA)

A

^{68}Ga labeling

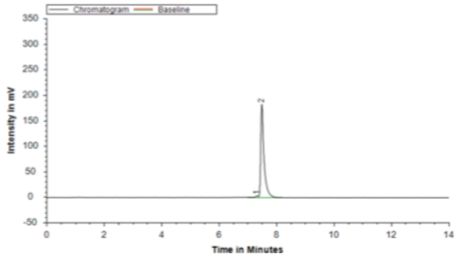

Result Table

No.	Ret. Time	Height	Area	Percent
1	7.316667	3.024592	20107.98	1.372243
2	7.486667	182.4775	1445229	98.62775

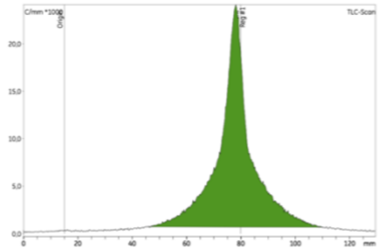

Integration TLC-Scan

Substance	R/F	%Total %	Type	Area Counts	%Area %
Reg #1	0,973	80,64	BB(M)	289581,9	100,00

B

^{177}Lu labeling

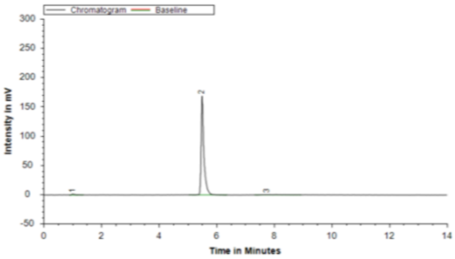

Result Table

No.	Ret. Time	Height	Area	Percent
1	1.003333	1.719583	13535.73	1.200401
2	5.486667	168.7589	1094893	97.09933
3	7.75	0.5924927	19172.21	1.700266

Figure S22: **A**: Analytical radio HPLC and TLC of [^{68}Ga]Ga-PS9; **B**: Analytical radio HPLC [^{177}Lu]Lu-PS9.

Supp. Fig. 23

PS – 10 (EuK-2Nal-HHH-ChX-DOTA)

A

^{68}Ga labeling

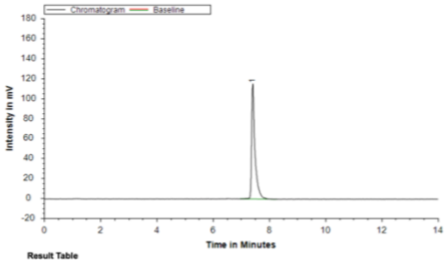

Result Table

No.	Ret. Time	Height	Area	Percent
1	7.41	115.2636	938572.2	100

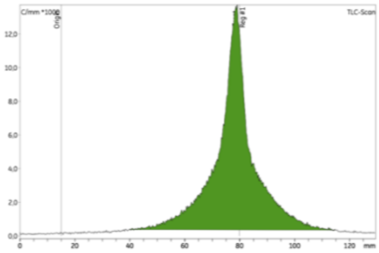

Integration TLC-Scan

Substance	R/F	%Total %	Type	Area Counts	%Area %
Reg #1	0,981	82,33	BB(M)	174059,6	100,00

B

^{177}Lu labeling

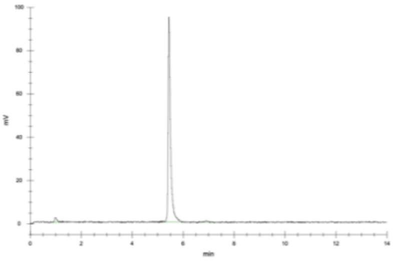

Nr.	Peak-Start (min)	Peak-Ende (min)	Ret. Zeit (min)	Fläche	Höhe (mV)	Prozent
1	0.810	1.320	0.987	17756.3	2.25	2.844
2	5.033	6.127	5.437	600715.3	94.54	96.200
3	6.520	7.263	6.927	5970.0	0.71	0.956

Figure S23: **A**: Analytical radio HPLC and TLC of [^{68}Ga]Ga-PS10; **B**: Analytical radio HPLC [^{177}Lu]Lu-PS10.

Supp. Fig. 24

PS – 11 (EuK-2Nal-ChX-HHH-DOTA)

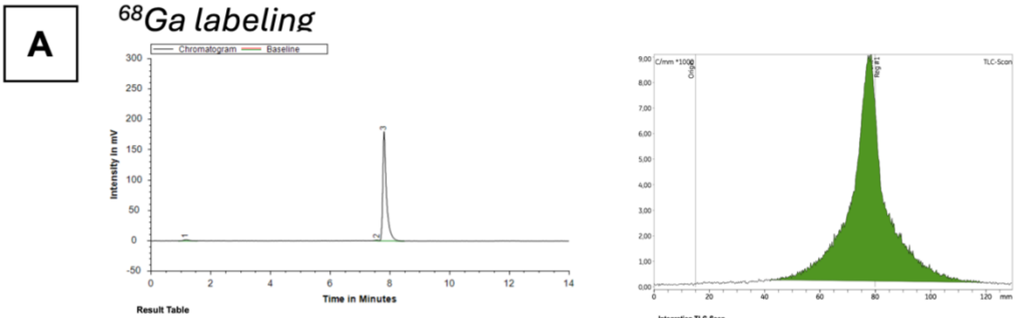

Result Table

No.	Ret. Time	Height	Area	Percent
1	1.156667	1.883588	21820.71	1.397664
2	7.573333	1.183411	9493.361	0.6080706
3	7.803333	180.1465	1529913	97.99426

Integration TLC-Scan

Substance	R/F	%Total %	Type	Area Counts	%Area %
Reg #1	0,973	83,29	BB(M)	122048,0	100,00

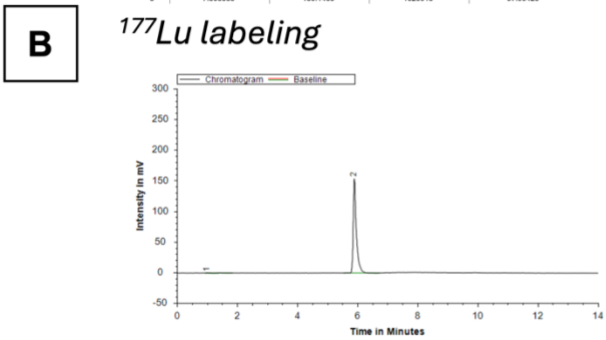

Result Table

No.	Ret. Time	Height	Area	Percent
1	0.9866667	0.6046564	7410.04	0.6667953
2	5.88	153.6228	1103882	99.33321

Figure S24: **A**: Analytical radio HPLC and TLC of [^{68}Ga]Ga-PS11; **B**: Analytical radio HPLC [^{177}Lu]Lu-PS11.

[^{68}Ga]Ga-PSMA-617

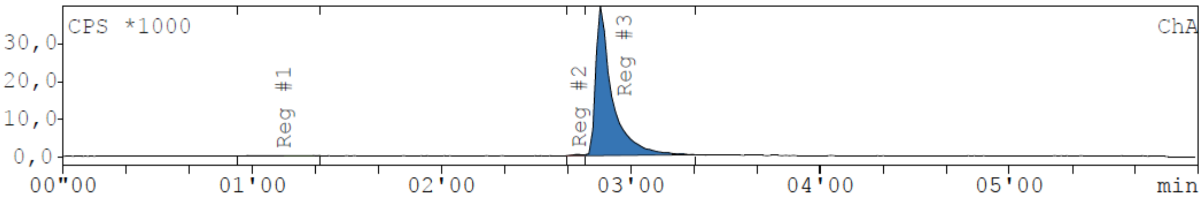

Integration ChA

Substanz	Ret s	Typ	Fläche Counts	%Fläche %
Reg #1	01'08	BB(M	1102,1	0,46
Reg #2	02'42	BD(M	913,8	0,38
Reg #3	02'52	DB(M	236028,6	99,15

[^{177}Lu]Lu-PSMA-617

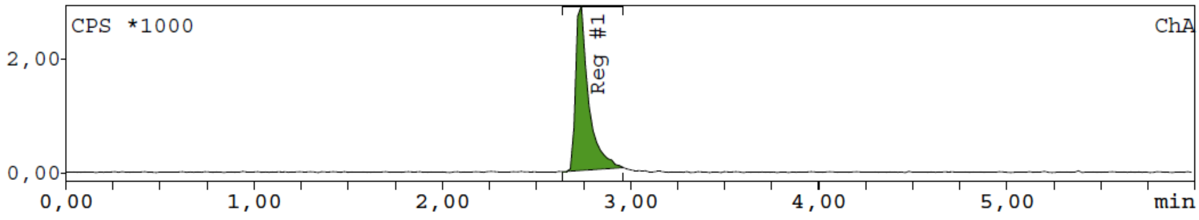

Integration ChA

Substanz	Ret min	Typ	Fläche Counts	%Fläche %
Reg #1	2,74	BB(M	13679,01	100,00

Figure S25: Analytical radio HPLCs of [^{68}Ga]Ga-PSMA-617 and [^{177}Lu]Lu-PSMA-617.

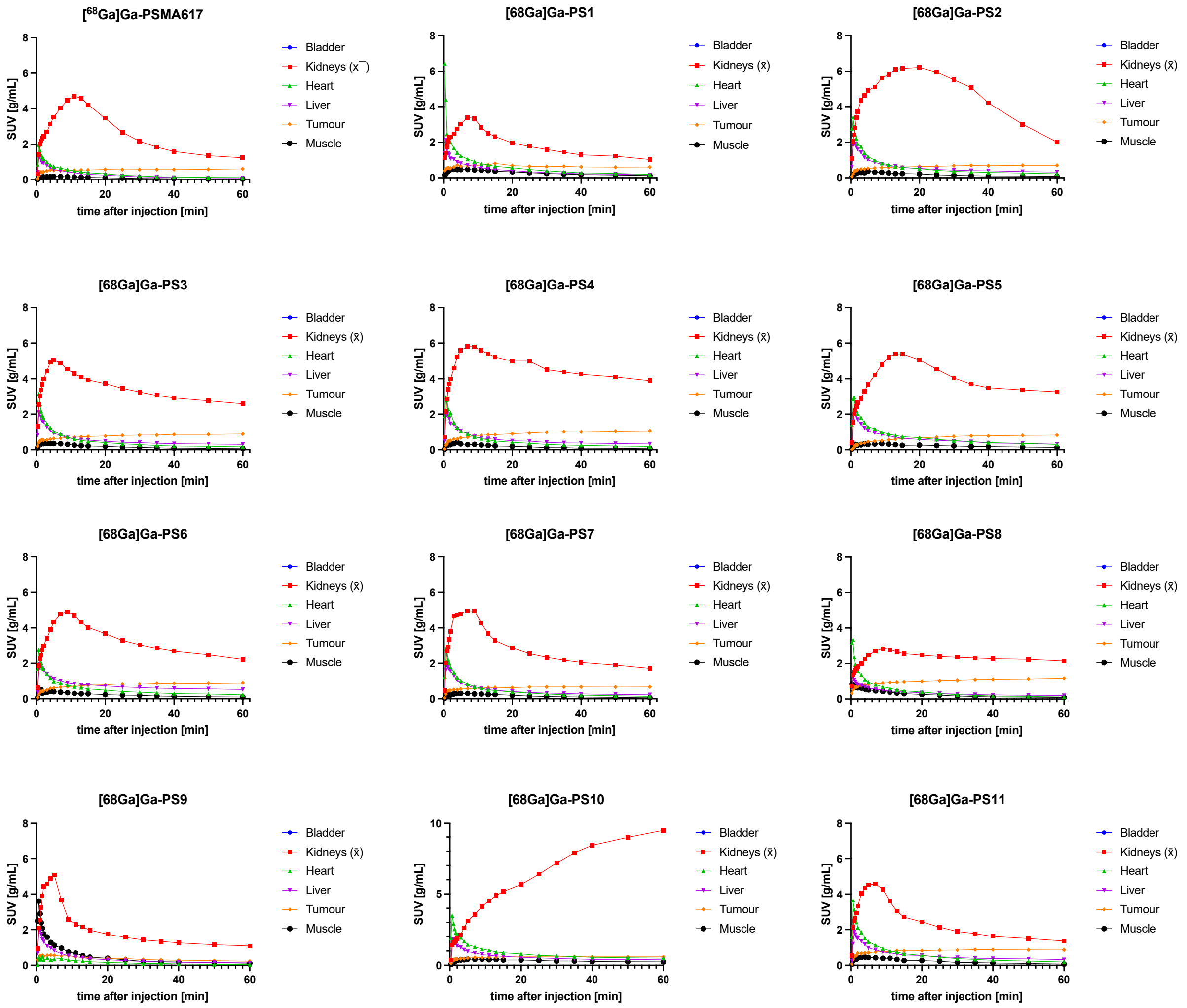

Figure S26: Time-activity curves (0 - 60 min) of PET scans from ^{68}Ga-labeled compounds showing accumulations in organs of interest in LNCaP tumor-bearing BALB/c nu/nu mice. SUV values of the two kidneys are displayed as an average.

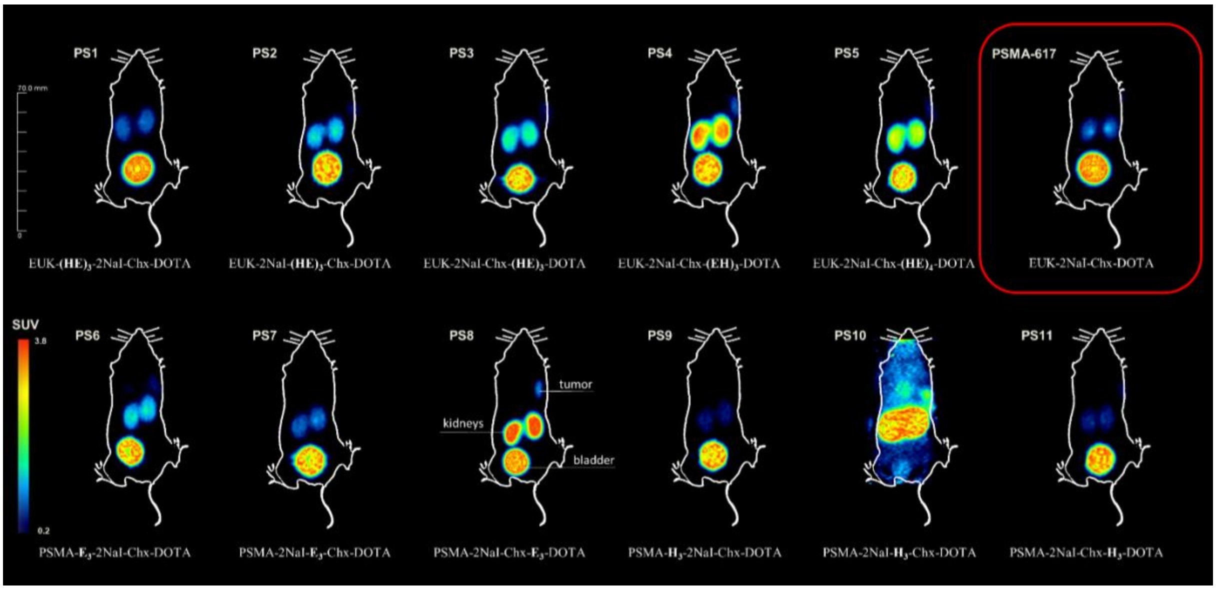

Figure S27: Maximum intensity projection from ^{68}Ga-labeled compounds 1 h p.i. of 0.5 nmol compound in LNCaP tumor-bearing BALB/c nu/nu mice.

S4. Supplementary Tables

Table S1: *p*-values of the Partition Coefficient (logD) of the ^{68}Ga-labeled compounds.

	PS1	PS2	PS3	PS4	PS5	PS6	PS7	PS8	PS9	PS10	PS11	PSMA-617
**PS1**	-	0.614	0.701	0.192	0.073	0.313	0.230	0.016	0.156	0.613	0.279	0.013
**PS2**	-	-	0.514	0.119	0.193	0.514	0.666	0.058	0.509	0.598	0.413	0.016
**PS3**	-	-	-	0.024	0.438	0.244	0.286	0.061	0.268	0.713	0.798	0.024
**PS4**	-	-	-	-	0.532	0.140	0.105	0.024	0.117	0.810	0.463	0.010
**PS5**	-	-	-	-	-	0.094	0.006	0.009	0.017	0.982	0.343	0.009
**PS6**	-	-	-	-	-	-	0.383	0.699	0.565	0.072	0.094	0.302
**PS7**	-	-	-	-	-	-	-	0.091	0.205	0.336	0.027	0.043
**PS8**	-	-	-	-	-	-	-	-	0.224	0.229	0.043	0.011
**PS9**	-	-	-	-	-	-	-	-	-	0.271	0.001	0.087
**PS10**	-	-	-	-	-	-	-	-	-	-	0.798	0.152
**PS11**	-	-	-	-	-	-	-	-	-	-	-	0.031
**PSMA-617**	-	-	-	-	-	-	-	-	-	-	-	-

Table S2: Stability in mouse serum of the ^{177}Lu-labeled compounds.

Time	PS1	PS2	PS3	PS4	PS5	PS6	PS7	PS8	PS9	PS10	PS11
[h]	[% intact substrate]										
**0**	100	100	100	100	100	100	100	100	100	100	100
**6**	97	100	100	100	100	100	100	100	81	97	100
**24**	95	98	100	100	100	96	100	100	61	91	100
**48**	91	96	100	100	100	94	100	100	39	64	100
**72**	85	93	98	100	97	94	100	100	35	63	100

Table S3: Stability in human serum of the ^{177}Lu-labeled compounds.

Time	PS1	PS2	PS3	PS4	PS5	PS6	PS7	PS8	PS9	PS10	PS11
[h]	[% intact substrate]										
**0**	100	100	100	100	100	100	100	100	100	100	100
**6**	97	99	100	100	100	100	100	100	98	100	100
**24**	95	98	100	100	100	100	100	100	90	98	100
**48**	88	94	100	100	98	100	100	100	80	91	100
**72**	80	90	100	100	99	100	100	100	78	85	100

Table S4: *p*-values of the compounds of the Competitive Cell Binding Assays.

	PS1	PS2	PS3	PS4	PS5	PS6	PS7	PS8	PS9	PS10	PS11	PSMA-617
**PS1**	-	0.11	0.03	0.0016	0.64	0.0064	0.041	<0.0001	0.35	0.86	0.0018	0.0008
**PS2**	-	-	0.49	0.057	0.88	0.12	0.066	0.0012	0.24	0.58	0.038	0.022
**PS3**	-	-	-	0.22	0.63	0.33	0.15	0.004	0.12	0.12	0.11	0.0079
**PS4**	-	-	-	-	0.36	0.91	0.50	0.0035	0.063	0.062	0.36	0.22
**PS5**	-	-	-	-	-	0.46	0.35	0.10	0.31	0.78	0.33	0.31
**PS6**	-	-	-	-	-	-	0.52	0.093	0.12	0.12	0.39	0.26
**PS7**	-	-	-	-	-	-	-	0.048	0.092	0.083	0.92	0.80
**PS8**	-	-	-	-	-	-	-	-	0.022	0.0080	0.019	0.0043
**PS9**	-	-	-	-	-	-	-	-	-	0.42	0.087	0.082
**PS10**	-	-	-	-	-	-	-	-	-	-	0.068	0.575
**PS11**	-	-	-	-	-	-	-	-	-	-	-	0.84
**PSMA-617**	-	-	-	-	-	-	-	-	-	-	-	-

Table S5: Surface Binding and Internalization of the ^{68}Ga-labeled compounds. Specific Surface Binding and Internalization were calculated by subtracting the unblocked values from the blocked values for each substance. Values are expressed as mean ± standard deviation.

	Surface Binding	Surface Binding Block	Internalization	Internalization Block	Specific Surface Binding	Specific Internalization
	[%Binding / %Internalization]					
**PS1**	1.15 ± 0.17	0.069 ± 0.012	0.66 ± 0.14	0.314 ± 0.024	1.09 ± 0.34	0.35 ± 0.3
**PS2**	1.01 ± 0.14	0.183 ± 0.024	0.55 ± 0.04	0.21 ± 0.036	0.83 ± 0.27	0.34 ± 0.24
**PS3**	1.32 ± 0.11	0.069 ± 0.129	0.67 ± 0.14	0.112 ± 0.022	1.26 ± 1.97	0.56 ± 0.42
**PS4**	1.43 ± 0.12	0.080 ± 0.009	0.81 ± 0.29	0.149 ± 0.047	1.35 ± 0.21	0.66 ± 0.68
**PS5**	1.41 ± 0.10	0.082 ± 0.029	0.67 ± 0.06	0.155 ± 0.022	1.33 ± 0.42	0.51 ± 0.25
**PS6**	0.76 ± 0.15	0.049 ± 0.02	0.60 ± 0.13	0.092 ± 0.032	0.71 ± 0.59	0.51 ± 0.58
**PS7**	0.64 ± 0.08	0.060 ± 0.021	0.40 ± 0.08	0.094 ± 0.025	0.58 ± 0.47	0.31 ± 0.49
**PS8**	0.99 ± 0.11	0.055 ± 0.011	0.71 ± 0.26	0.092 ± 0.032	0.94 ± 0.33	0.62 ± 0.72
**PS9**	0.94 ± 0.02	0.037 ± 0.009	0.59 ± 0.06	0.082 ± 0.005	0.90 ± 0.28	0.51 ± 0.17
**PS10**	0.57 ± 0.04	0.027 ± 0.005	0.51 ± 0.07	0.205 ± 0.020	0.55 ± 0.29	0.31 ± 0.24
**PS11**	0.81 ± 0.04	0.123 ± 0.015	0.66 ± 0.09	0.077 ± 0.009	0.69 ± 0.17	0.58 ± 0.26
**PSMA-617**	0.79 ± 0.13	0.062 ± 0.013	0.50 ± 0.05	0.210 ± 0.036	0.72 ± 0.38	0.28 ± 0.27

Table S6: *p*-values of the ^{68}Ga-labeled compounds of the Surface Binding Assay.

	PS1	PS2	PS3	PS4	PS5	PS6	PS7	PS8	PS9	PS10	PS11	PSMA-617
**PS1**	-	0.32	0.22	0.073	0.081	0.029	0.0021	0.19	0.057	0.0031	0.0254	0.0088
**PS2**	-	-	0.044	0.019	0.0080	0.10	0.0070	0.85	0.33	0.0066	0.0759	0.056
**PS3**	-	-	-	0.34	0.35	0.0072	0.0003	0.016	0.0014	0.0005	0.0023	0.0011
**PS4**	-	-	-	-	0.83	0.0043	0.0002	0.0056	0.0006	0.0004	0.0015	0.0005
**PS5**	-	-	-	-	-	0.0011	<0.0001	0.0021	0.0002	<0.0001	0.0003	0.0001
**PS6**	-	-	-	-	-	-	0.23	0.072	0.066	0.10	0.63	0.85
**PS7**	-	-	-	-	-	-	-	0.0031	0.0006	0.25	0.028	0.11
**PS8**	-	-	-	-	-	-	-	-	0.41	0.0024	0.058	0.042
**PS9**	-	-	-	-	-	-	-	-	-	<0.0001	0.0068	0.0545
**PS10**	-	-	-	-	-	-	-	-	-	-	0.0034	0.039
**PS11**	-	-	-	-	-	-	-	-	-	-	-	0.75
**PSMA-617**	-	-	-	-	-	-	-	-	-	-	-	-

Table S7: *p*-values of the ^{68}Ga-labeled compounds of the Internalization Assay.

	PS1	PS2	PS3	PS4	PS5	PS6	PS7	PS8	PS9	PS10	PS11	PSMA-617
**PS1**	-	0.27	0.93	0.42	0.94	0.61	0.025	0.76	0.43	0.19	0.99	0.051
**PS2**	-	-	0.24	0.21	0.049	0.56	0.049	0.36	0.37	0.54	0.12	0.18
**PS3**	-	-	-	0.51	0.96	0.59	0.030	0.83	0.38	0.18	0.95	0.046
**PS4**	-	-	-	-	0.38	0.33	0.045	0.66	0.20	0.17	0.46	0.051
**PS5**	-	-	-	-	-	0.44	0.0034	0.76	0.17	0.040	0.96	0.0036
**PS6**	-	-	-	-	-	-	0.068	0.55	0.90	0.40	0.53	0.16
**PS7**	-	-	-	-	-	-	-	0.072	0.014	0.14	0.013	0.087
**PS8**	-	-	-	-	-	-	-	-	0.42	0.28	0.79	0.12
**PS9**	-	-	-	-	-	-	-	-	-	0.21	0.27	0.043
**PS10**	-	-	-	-	-	-	-	-	-	-	0.041	0.68
**PS11**	-	-	-	-	-	-	-	-	-	-	-	0.014
**PSMA-617**	-	-	-	-	-	-	-	-	-	-	-	-

Table S8: SUV values of the ^{68}Ga-labeled compounds 1 h and 2 h p.i. of the relevant organs in PET imaging in LNCaP tumor-bearing BALB/c nu/nu mice. Values are expressed as mean ± standard deviation due to the VOIs drawn in PMOD.

Abbr.	tumor [g/ml]		kidneys [g/ml]		muscle [g/ml]	
	**1 h**	**2h**	**1 h**	**2h**	**1 h**	**2h**
**PSMA-617**	0.63 ± 0.02	0.52 ± 0.16	1.25 ± 0.14	0.28 ± 0.06	0.073 ± 0.004	0.02 ± 0.01
**PS1**	0.43 ± 0.06	0.30 ± 0.11	1.71 ± 0.20	1.32 ± 0.15	0.09 ± 0.01	0.08 ± 0.02
**PS2**	0.78 ± 0.05	0.74 ± 0.06	1.64 ± 0.10	1.01 ± 0.09	0.11 ± 0.02	0.11 ± 0.01
**PS3**	0.93 ± 0.08	0.92 ± 0.06	3.05 ± 0.01	1.56 ± 0.13	0.13 ± 0.03	0.1 ± 0.01
**PS4**	1.15 ± 0.04	1.07 ± 0.09	3.81 ± 0.15	2.77 ± 0.20	0.11 ± 0.02	0.1 ± 0.03
**PS5**	0.86 ± 0.06	0.94 ± 0.07	3.13 ± 0.16	2.37 ± 0.27	0.12 ± 0.02	0.09 ± 0.02
**PS6**	0.69 ± 0.07	0.85 ± 0.08	1.53 ± 0.08	0.40 ± 0.05	0.11 ± 0.02	0.11 ± 0.02
**PS7**	0.71 ± 0.05	0.56 ± 0.08	1.60 ± 0.12	0.59 ± 0.06	0.09 ± 0.01	0.12 ± 0.02
**PS8**	1.32 ± 0.08	0.86 ± 0.39	3.89 ± 0.65	2.70 ± 0.89	0.08 ± 0.02	0.11 ± 0.02
**PS9**	0.21 ± 0.05	0.12 ± 0.03	0.98 ± 0.09	0.74 ± 0.09	0.082 ± 0.002	0.07 ± 0.03
**PS10**	0.65 ± 0.04	0.37 ± 0.05	8.13 ± 1.31	1.09 ± 0.06	0.21 ± 0.03	0.06 ± 0.02
**PS11**	0.82 ± 0.07	0.88 ± 0.09	1.24 ± 0.08	0.77 ± 0.13	0.11 ± 0.02	0.10 ± 0.03
